# Author Correction: Early response evaluation by single cell signaling profiling in acute myeloid leukemia

**DOI:** 10.1038/s41467-023-37488-8

**Published:** 2023-03-30

**Authors:** Benedicte Sjo Tislevoll, Monica Hellesøy, Oda Helen Eck Fagerholt, Stein-Erik Gullaksen, Aashish Srivastava, Even Birkeland, Dimitrios Kleftogiannis, Pilar Ayuda-Durán, Laure Piechaczyk, Dagim Shiferaw Tadele, Jørn Skavland, Panagotis Baliakas, Randi Hovland, Vibeke Andresen, Ole Morten Seternes, Tor Henrik Anderson Tvedt, Nima Aghaeepour, Sonia Gavasso, Kimmo Porkka, Inge Jonassen, Yngvar Fløisand, Jorrit Enserink, Nello Blaser, Bjørn Tore Gjertsen

**Affiliations:** 1 Centre for Cancer Biomarkers (CCBIO), Department of Clinical Science, University of Bergen, Bergen, Norway; 2 Department of Internal Medicine, Hematology Section, Haukeland University Hospital, Helse Bergen HF, Bergen, Norway; 3 Genome Core Facility, Clinical Laboratory, K2 Haukeland University Hospital, Bergen, Norway; 4 The Proteomics Facility of the University of Bergen (PROBE), University of Bergen, Bergen, Norway; 5 Centre for Cancer Biomarkers and Computational Biology Unit, Department of Informatics, University of Bergen, Bergen, Norway; 6 Department of Molecular Cell Biology, Institute for Cancer Research, The Norwegian Radium Hospital, Montebello, 0379 Oslo Norway; 7 Centre for Cancer Cell Reprogramming, Institute of Clinical Medicine, Faculty of Medicine, University of Oslo, 0318 Oslo, Norway; 8 Faculty of Medicine, University of Oslo, Oslo, Norway; 9 Department of Molecular Genetics, Division of Laboratory Medicine, Oslo University Hospital, Oslo, Norway; 10 Department of Translational Hematology and Oncology Research, Cleveland Clinic, OH 44106 USA; 11 Department of Immunology, Genetics and Pathology, Science for Life Laboratory, Uppsala University, Uppsala, Sweden; 12 Center for Medical Genetics and Molecular Medicine, Haukeland University Hospital, Bergen, Norway; 13 Department of Pharmacy, UiT-The Arctic University of Norway, 9037 Tromsø, Norway; 14 Department of Hematology, Oslo University Hospital, Oslo, Norway; 15 Department of Anesthesiology, Perioperative and Pain Medicine, Stanford University School of Medicine, Stanford, CA 94121 USA; 16 Department of Pediatrics, Stanford University School of Medicine, Stanford, CA 94121 USA; 17 Department of Biomedical Informatics, Stanford University School of Medicine, Stanford, CA 94121 USA; 18 Centre for Clinical Treatment Research (NeuroSysMed), Department of Clinical Science, University of Bergen, Bergen, Norway; 19 Department of Hematology, Helsinki University Hospital Comprehensive Cancer Center, Helsinki, Finland; 20 Section for Biochemistry and Molecular Biology, Faculty of Mathematics and Natural Sciences, University of Oslo, 0037 Oslo, Norway; 21 Department of Informatics, University of Bergen, Bergen, Norway

**Keywords:** Acute myeloid leukaemia, Predictive markers, Translational research, Stress signalling

Correction to: *Nature Communications* 10.1038/s41467-022-35624-4, published online 07 January 2023

In the original version of this article, the given and family names of Panagotis Baliakas were incorrectly structured.

The original article has been corrected.

