# Supplementary material for: Early response evaluation by single cell signaling profiling in acute myeloid leukemia
Source: Nat Commun. 2023 Jan 7;14:115. doi: 10.1038/s41467-022-35624-4 (PMC9825407; doi:10.1038/s41467-022-35624-4)
Supplement: Supplementary file 1 — Supplementary information [file 41467_2022_35624_MOESM1_ESM.pdf]

Supplementary information

Early response evaluation by single cell signaling profiling in acute myeloid leukemia

Tislevoll et al. 2022

Supplementary Table 1

Patient Characteristics

Patient nr	*Age*	*WBC*	*Diagnosis*	*De novo/ sAML*	*FAB*	*ELN 2017 risk*	*HOVON 132*	*Treatment*	*CR/ nonCR*	*TX status*	*5-y-OS (Days)*	*24h-pERK1/2 Group MC9*	*MRD*
P1	35-60	2.7	AML	De novo	M2	Intermediate	NA	ST	CR	Allo	Alive	Low	NA
P2	>60	2.6	AML	De novo	M4	Favorable	NA	ST	CR	NA	Alive	Low	NA
P3	<35	0.6	AML	De novo	M5	Adverse	NA	ST	CR	Allo	Alive	Low	NA
P4	>60	3.5	AML	De novo	M5	Adverse	NA	ST	nonCR	Allo	456	High	NA
P5	35-60	34.0	AML	De novo	M4/M5	Adverse	Included	ST+Len	CR	Allo	Alive	Low	negative
P6	>60	33.7	AML	De novo	M4	Favorable	Included	ST + Len	CR	Auto	Alive	Low	negative
P7	>60	2.0	AML	De novo	M4	Favorable	Included	ST + Len	Aplastic	Auto	246	High	positive
P8	35-60	8.4	Biphenotypic AML/BALL	De novo	M1/M2	Intermediate	Included	ST+ Len	CR	Allo	189	High	Positive
P9	<35	150.6	AML	De novo	M5	Adverse	Included	ST	nonCR	Allo	286	High	negative
P10	>60	1.4	AML	De novo	M2	Intermediate	Included	ST + Len	CR	Allo	Alive	Low	No LAIPs
P11	>60	12.3	AML	sAML	M5	Adverse	NA	ST	Aplastic	NA	328	High	NA
P12	>60	3.5	AML	sAML	NA	Intermediate	NA	ST	non CR	Allo	502	High	NA
P13	>60	30.0	AML	De novo	M1/M2	Favorable	Included	ST	CR	Allo	Alive	Low	negative MRD, positive NPM1 MRD
P14	<35	34.1	AML	De novo	M4/M5	Intermediate	Included	ST	CR	Auto	448	High	negative
P15	>60	95.6	AML	De novo	M1	Intermediate	NA	ST	nonCR	NA	378	High	NA
P16	>60	77.5	AML	De novo	M2	Favorable	NA	ST	CRi	NA	221	Low	NA
P17	>60	3.7	AML	De novo	M4/M5	Adverse	NA	ST	CR	NA	56	High	NA
P18	35-60	3.8	AML	De novo	NA	Adverse	Included	ST+ Len	nonCR	Allo	Alive	Low	negative
P19	>60	1.4	AML	De novo	M0/M1	Intermediate	NA	ST	nonCR	NA	51	Low	NA
P20	35-60	13.7	AML	De novo	M2	Adverse	Included	ST + Len	CR	Allo	Alive	High	negative
P21	35-60	4.7	AML	De novo	M4	Favorable	Included	ST+ Len	Aplastic	NA	24	High	NA
P22	35-60	11.4	AML	De novo	NA	Favorable	Included	ST	CR	NA	324	Low	negative
P23	>60	22.6	AML	De novo	M4	Favorable	Included	ST + Len	Aplastic	Auto	447	High	positive
P24	35-60	55.9	AML	De novo	M2	Favorable	Included	ST	CR	NA	1048	High	negative
P25	>60	2.0	AML	De novo	M1	Intermediate	Included	ST	nonCR	Allo	229	Low	NA
P26	>60	7.5	AML	De novo	M2	Adverse	NA	ST	nonCR	NA	162	High	NA
P27	>60	17.8	AML	De novo	M0	Adverse	NA	ST	nonCR	Allo	321	High	NA
P28	35-60	2.6	AML	De novo	M1	Adverse	Included	ST	CRi	NA	48	Low	NA
P29	>60	0.7	AML	De novo	M0	Intermediate	Included	ST+ Len	Aplastic	Allo	Alive	Low	negative
P30	>60	270.6	AML	De novo	M5	Favorable	NA	ST	CR	NA	Alive	Low	NA
P31	35-60	0.8	AML	De novo	M1/M2	Adverse	NA	ST	CR	NA	Alive	Low	NA
P32	>60	11.8	AML	De novo	M1/M2	Favorable	Included	ST	Aplastic	NA	49	High	NA
P33	35-60	145.0	AML	De novo	M0/M1	Intermediate	NA	ST (-)	CR	Allo	457	NA	NA
P34	35-60	NA	AML	sAML	NA	Adverse	NA	ST (-)	Aplastic	NA	528	NA	NA

Abbreviations:
WBC: (White blood cell count): at diagnosis in x10^09/L
Age: presented in range: below 35y (<35), 35-60,
sAML: secondary AML
FAB: French-American-British (FAB) classification of AML
ELN Risk: European Leukemia Net (ELN) risk classification of AML
HOVON 132: Patients included in HOVON 132 clinical trial (Included)
Treatment: ST = Standard "7+3" induction therapy, ST+Len = addition of per-oral treatment with lenalidomide at days 1-21 in cycle 2 (HOVON 132),
ST (-) = dose reduced "7+3".
CR/nonCR: CR= complete remission, nonCR= >6% blasts in bone marrow prior to cycle 2.
TX- status: Allogeneic stem cell transplantation (Allo-HSCT), Auto: Autologous stem cell transplantation.
MRD: Minimal residual disease, measured after cycle 2.

Supplementary Table1. Patient characteristics. Clinical characteristics of the 34 AML patients included in our study.

Supplementary Table 2

Cohort summary

Characteristic	*All patients (n=32)*
Age (median) yr	56.30<br>Age range (16 -71yr)
**Age distribution**	
Under 60 yr	13
Over 60 yr	19
**Male**	19
**Female**	13
**ELN 2017 risk**	
Favorable	11
Intermediate	9
Adverse	12
**Complete remission (CR)**	
CR/CRi	17
Aplastic	6
non CR	9
**Transplantation**	
Allogeneic	14
Autologeous	4
**5y - Survival**	
Alive	12
Deceased	20
**Mutations**	
FLT3-ITD	6
NPM1	7
Inv(16)	6
TP53	2

Supplementary Table 2. Cohort summary. A summary of clinical characteristics of the 32 AML patients included in the data analysis in our study. P33 and P34 is not presented here as they were only included in one analysis, as described in the methods section.

Supplementary Table 3

Antibody panel

Metal	*Isotope*	*Antigen*	*Clone*	*Intracellular/ Surface*	*Vendor*	*Catalog #*	*Dilution*
Nd	142	Cleaved Caspase 3	D3E9	I	Fluidigm	3142004A	1:400
Sm	149	p4E-BP1 [T37/T46]	236B4	I	Fluidigm	3149005C	1:400
Nd	150	pSTAT5 [Y694]	47	I	Fluidigm	3150005A	1:400
Sm	152	pAkt [S473]	D9E	I	Fluidigm	3152005C	1:200
Eu	153	pSTAT1 [Y701]	58D6	I	Fluidigm	3153003C	1:400
Sm	154	pRB [S807/S811]	J112-906	I	BD BioScience	413568	1:400
Gd	156	pP38[T180/Y182]	D3F9	I	Fluidigm	3156002C	1:1600
Gd	158	pSTAT3 [Y705]	4/P-STAT3	I	Fluidigm	3158005C	1:400
Dy	161	pAxl [Y779]	Y779	I	R&D	MAB6965	1:200
Dy	164	CyclinB1	GNS-1	I	Fluidigm	3153009C	1:200
Ho	165	pCREB [S133]	87G3	I	Fluidigm	3165009C	1:200
Er	166	pNFkB p65 [S529]	K10-895.12.50	I	Fluidigm	3166006A	1:400
Er	167	pERK 1/2 [T202/Y204]	D13.14.4E	I	Fluidigm	3167005C	1:400
Yb	172	pS6 [S235/S236]	N7-548	I	Fluidigm	3172008C	1:400
Lu	175	pHistone3 [S28]	HTA28	I	Fluidigm	3175012A	1:200
Bi	209	CD11b (Mac-1)	ICRF44	S	Fluidigm	3209003B	1:1600
Dy	162	CD8a	RPA-T8	S	Fluidigm	3162015C	1:3200
Dy	163	CD33	WM53	S	Fluidigm	3163023B	1:800
Er	168	CD34	581	S	BioLegend	343531	1:12000
Er	170	CD3	UCHT1	S	Fluidigm	3170001B	1:3200
Eu	151	CD123 (IL-3R)	6H6	S	Fluidigm	3151001B	1:800
Gd	155	CD56 (NCAM)	B159	S	Fluidigm	3155008B	1:400
Gd	160	CD14	M5E2	S	BioLegend	301843	1:800
Nd	143	CD117 (c-kit)	104D2	S	Fluidigm	3143001C	1:1600
Nd	144	CD38	HIT2	S	Fluidigm	3144014C	1:800
Nd	145	CD4	RPA-T4	S	Fluidigm	3145001B	1:800
Nd	146	CD64	10.1	S	Fluidigm	3146006C	1:400

Nd	148	CD16	3G8	S	Fluidigm	3148004B	1:200
Pr	141	CD66b	G10F5	S	BioLegend	305102	1:3200
Sm	147	CD20	2H7	S	Fluidigm	3147001B	1:400
Tb	159	CD90 (Thy-1)	5E10	S	Fluidigm	3159007C	1:400
Tm	169	CD25 (IL-2R)	2A3	S	Fluidigm	3169003C	1:3200
Y	89	CD45	HI30	S	Fluidigm	3089003B	1:4000
Yb	171	Axl	1H12	S	BerGenBio AS	Provided by BerGenBio ASA	1:200
Yb	174	HLA-DR	L243	S	Fluidigm	3174001C	1:800
Yb	176	CD7	CD7-6B7	S	BioLegend	343107	1:3200

* Six antibodies, namely CD14, CD34, pRB, CD7, AXL and pAxl were conjugated in-house to the isotopes, using the Maxpar X8 antibody conjugation Kit as described by the manufacturer (Fluidigm).

Supplementary Table 3. Antibody panel. A description of the antibody panel used in this study.

Supplementary Figure 1

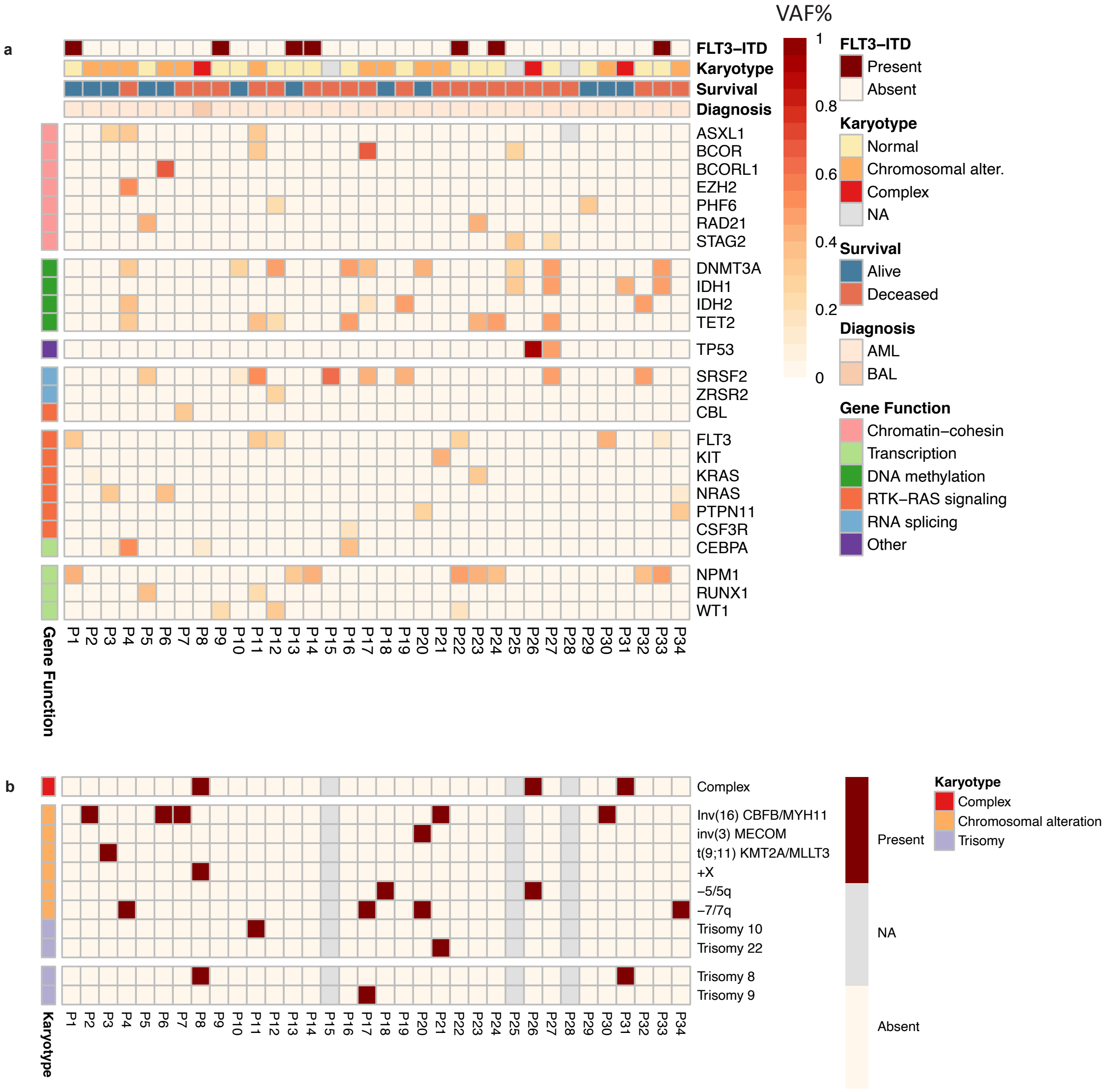

Supplementary Figure 1. Mutations and cytogenetics at diagnosis

a, Mutations identified among the 54 genes frequently mutated in myeloid malignancies sequenced by the Illuminas TruSight Myeloid Gene Panel for all 34 patients analyzed by mass cytometry in our study. Mutations are visualized as variant allele frequency (VAF%). Patient 28 had a ASXL mutation identified by the diagnostic cytogenetics workup performed when the patient was included in the HOVON SAKK 132 study, however, this mutation was not verified in our TruSight myeloid panel. P19 had a monoallelic CEBPA mutation by the diagnostic workup but was not verified by NGS. b, Diagnostic cytogenetics including G-banding, RT-PCR, FISH and fragment analysis of FLT3 and NPM1 displayed as present, NA or absent. Abbreviations: BAL: bi-phenotypic leukemia.

Supplementary Figure 2

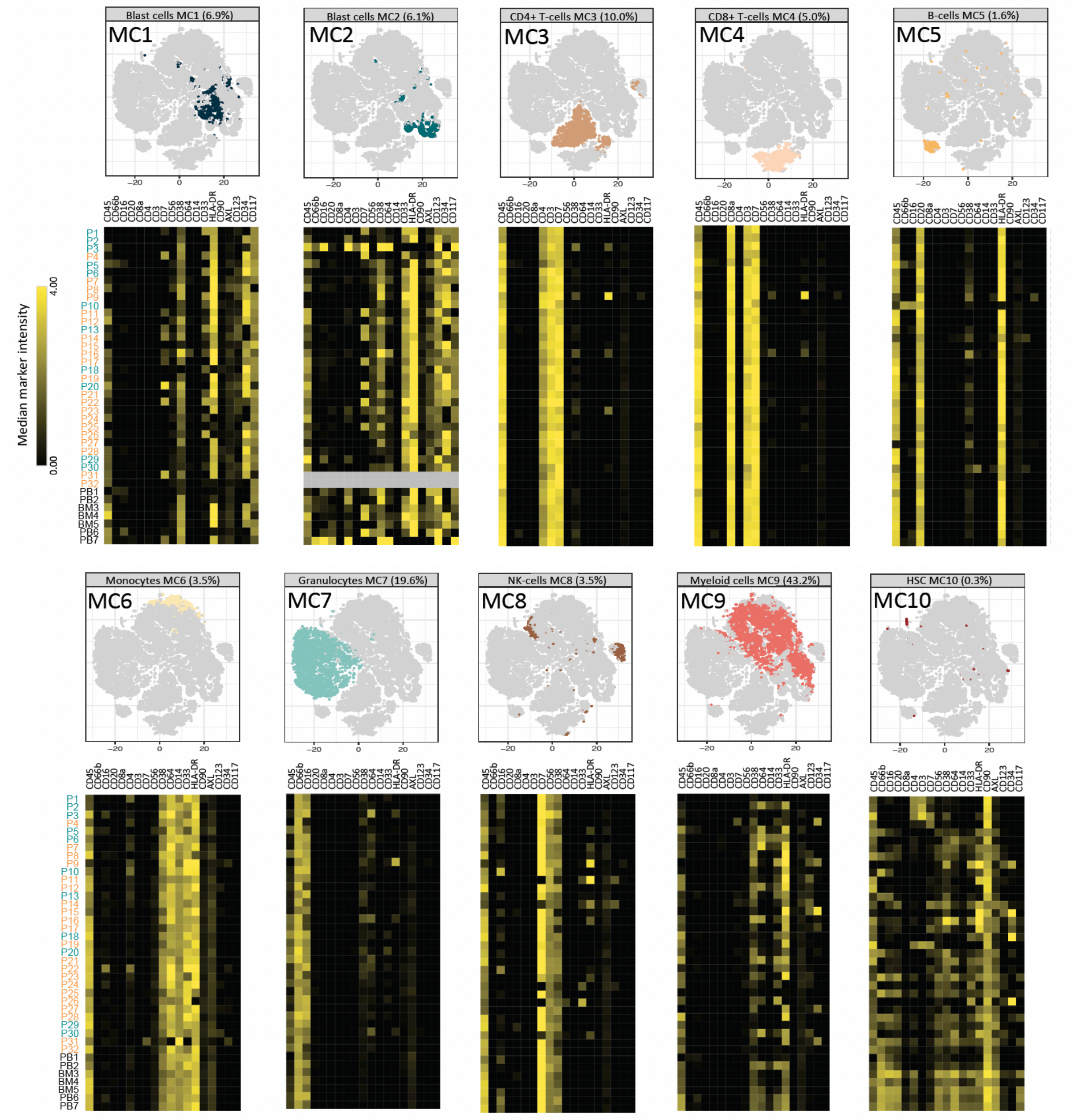

Supplementary Figure 2. Metacluster immunophenotype per patient t-SNE plots highlighting the position and distribution of metacluster 1 -10 (MC1-10) (color) in the t-SNE plot of all 32 patients at pre-treatment (grey). Heatmaps below the t-SNE plot show the median marker intensity for the surface markers in each metacluster for each patient. Patients are sorted by date of sampling and colored by patient 5-year survival (blue=alive, orange=deceased).

Supplementary Figure 3

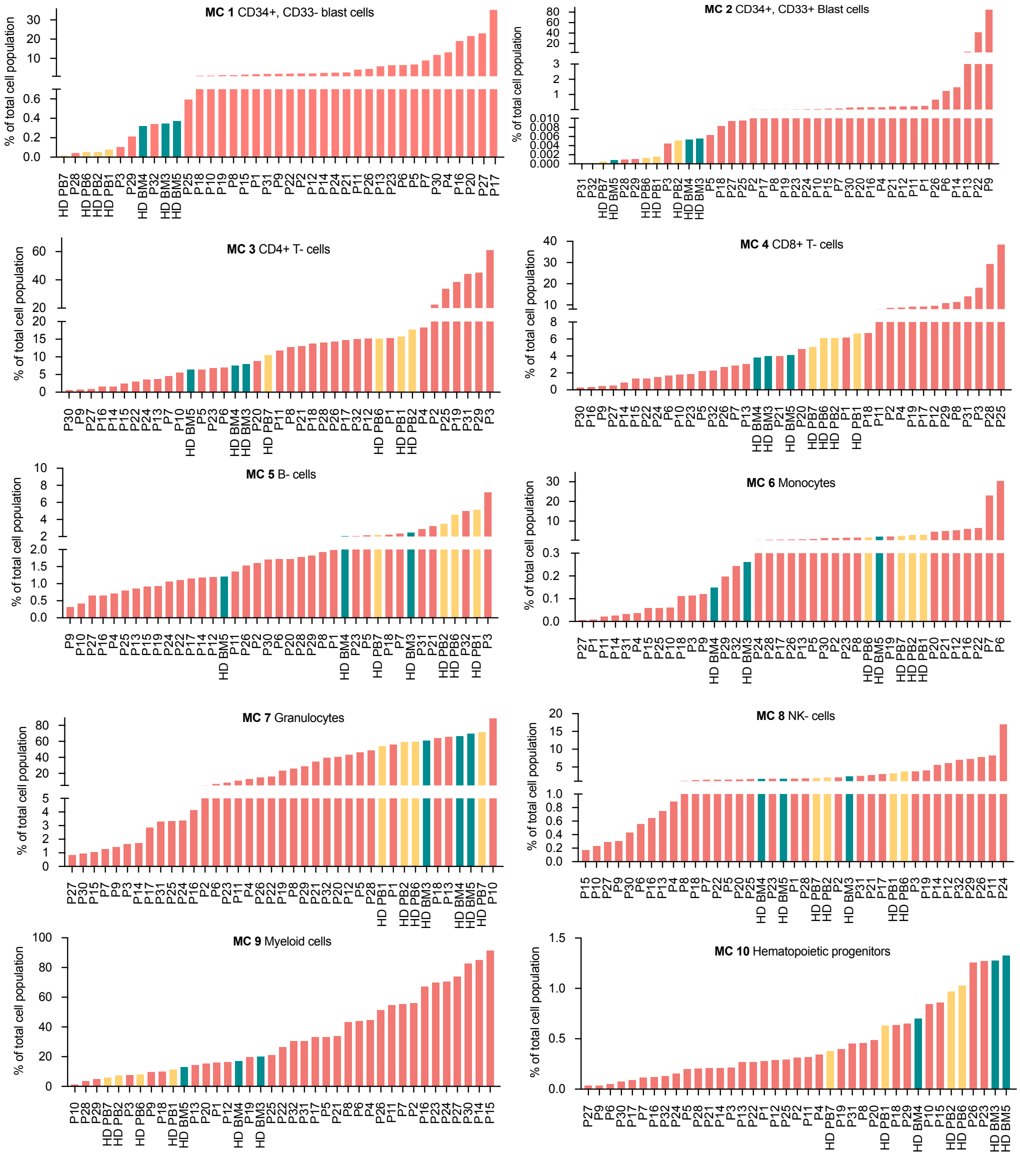

Supplementary Figure 3. Metacluster size per patient

Graphs showing the metacluster size in each patient and healthy donor (HD) as percent of total cell population. All patients are colored in red (n=32), healthy peripheral blood donors (HD PB) (n=4) in yellow and healthy bone marrow donors (HD BM) (n=3) in green.

Supplementary Figure 4

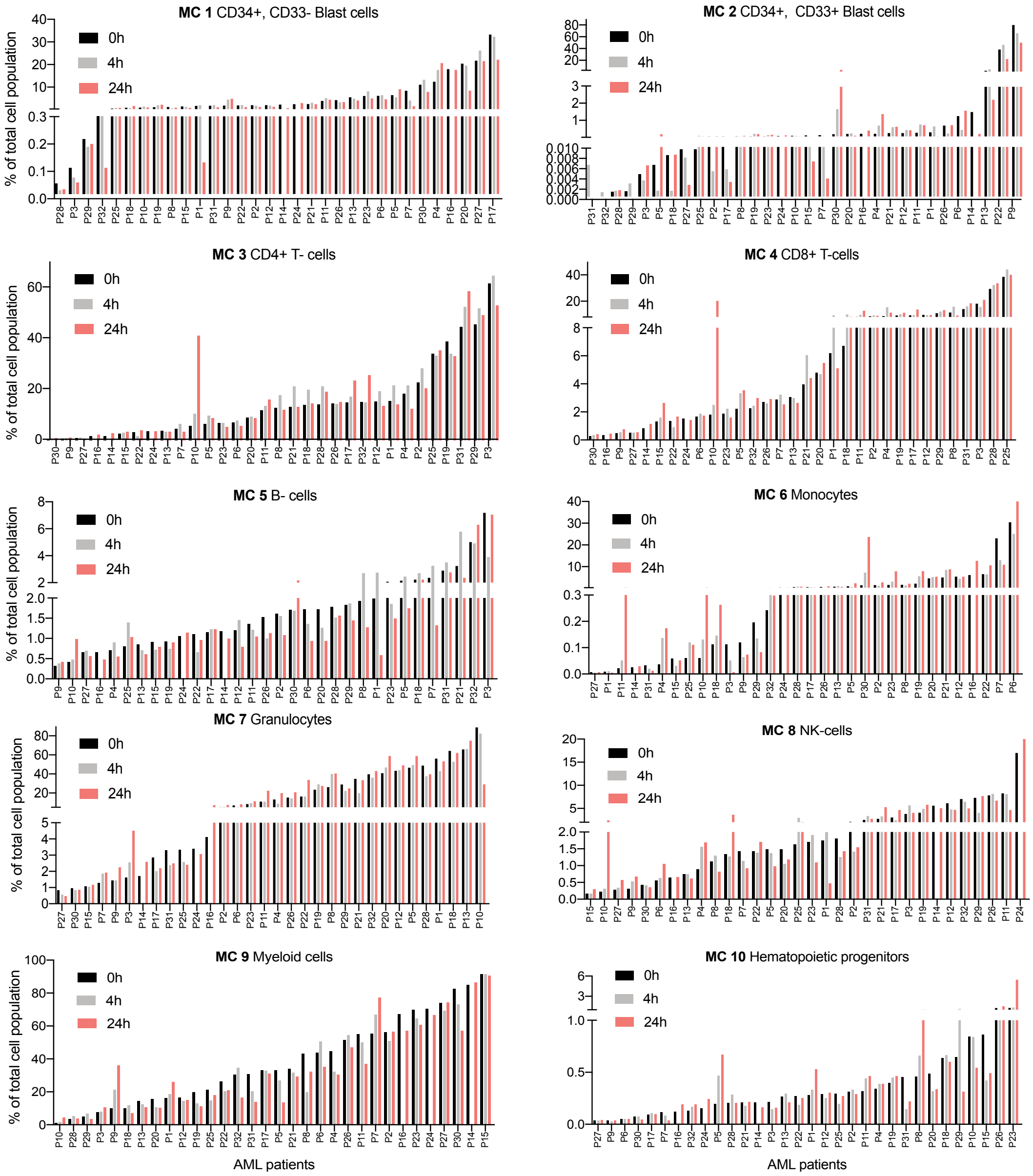

Supplementary Figure 4. Metacluster size per patient at all timepoints

Graphs showing the metacluster (MC) size in each patient at pre-treatment (0h- black), 4 hours (4h -grey) and 24 hours after start of chemotherapy (24h- red) as percent of total cell population.

Supplementary Figure 5

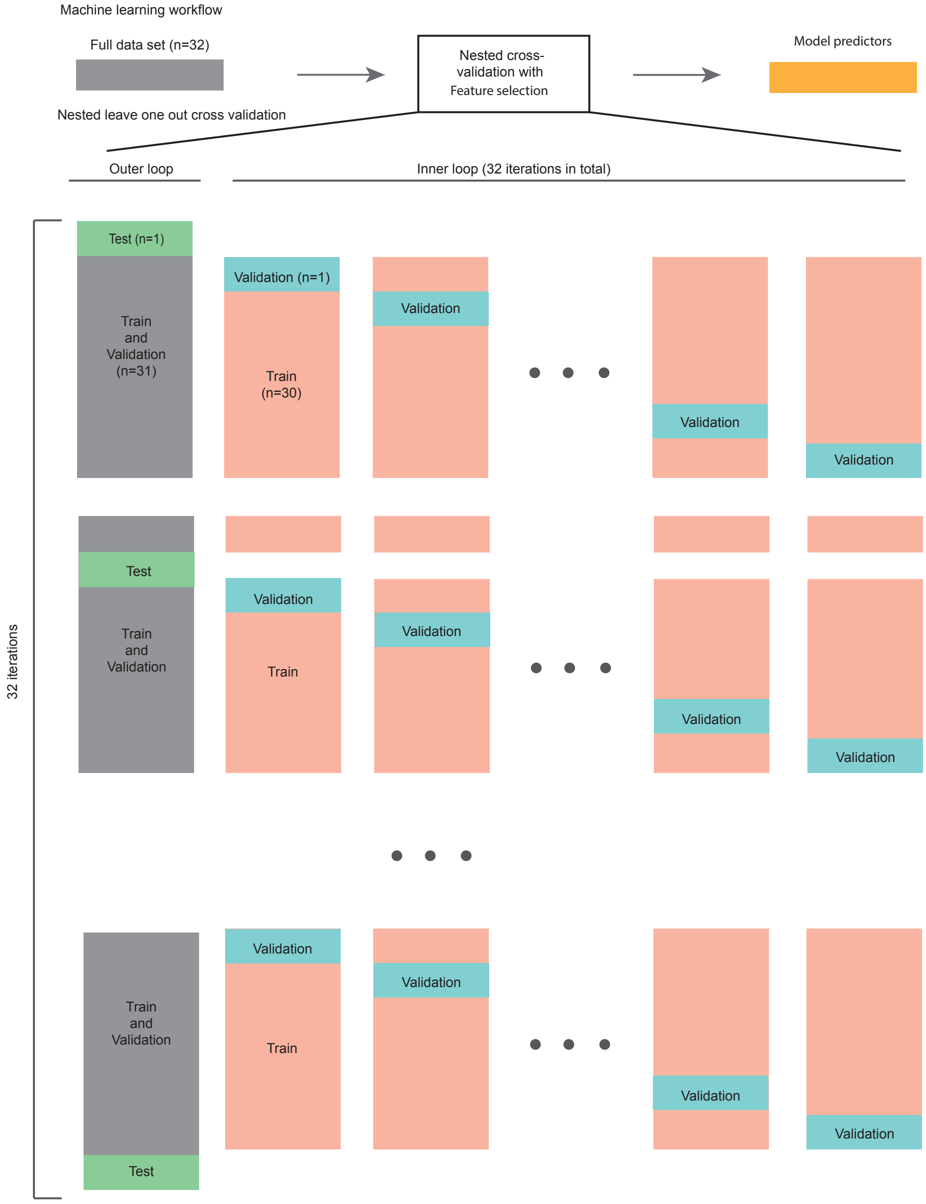

Supplementary Figure 5. Machine learning pipeline We used LASSO Cox regression analysis with nested leave-one-out cross-validation. Since we used leave-one-out cross-validation and only had 32 patients, we could use all possible 1-patient subsets as test samples, all 1-patient subsets as validation samples and all 30-patient subsets as training data.

Supplementary Figure 6

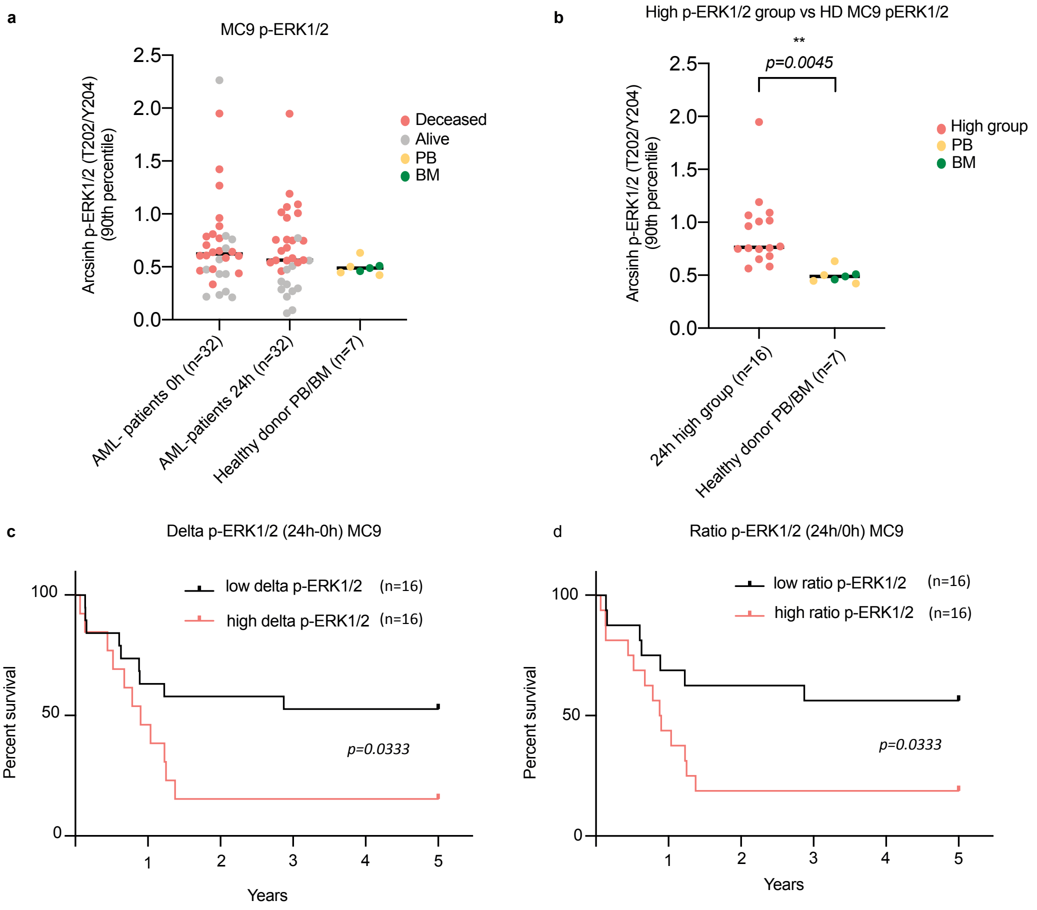

Supplementary Figure 6. p-ERK1/2 in MC9 in AML patients and healthy donors and pERK1/2 ratio from pre-treatment to 24 h

a, Arcsinh transformed 90th percentile p-ERK1/2 in metacluster (MC) 9 for all 32 AML patients at 0h and 24 h (5y-OS: red=deceased, grey=alive) and healthy donors (peripheral blood (PB) =yellow, bone marrow (BM) = green). b, Arcsinh transformed 90th percentile p-ERK1/2 in MC9 for patients in high p-ERK1/2 group (red) (n=16) and healthy donors (n=7). A two-tailed unpaired t-test was used to compare high group to healthy donors. c,d Kaplan-Meier survival curves of c, delta p-ERK1/2 (24h-0h) in MC9 devided into two groups, based on the median value, with 16 patients in each group. d, ratio p-ERK (24h/0h) in MC9 divided into two groups, based on the median value, with 16 patients in each group. p-values between survival curves are calculated using a Log-rank (Mantel-Cox) test (p=0.0333, HR (logrank) low/high group 0.3862 and 95% CI of ratio 0.15.88 to 0.9392).

Supplementary Figure 7

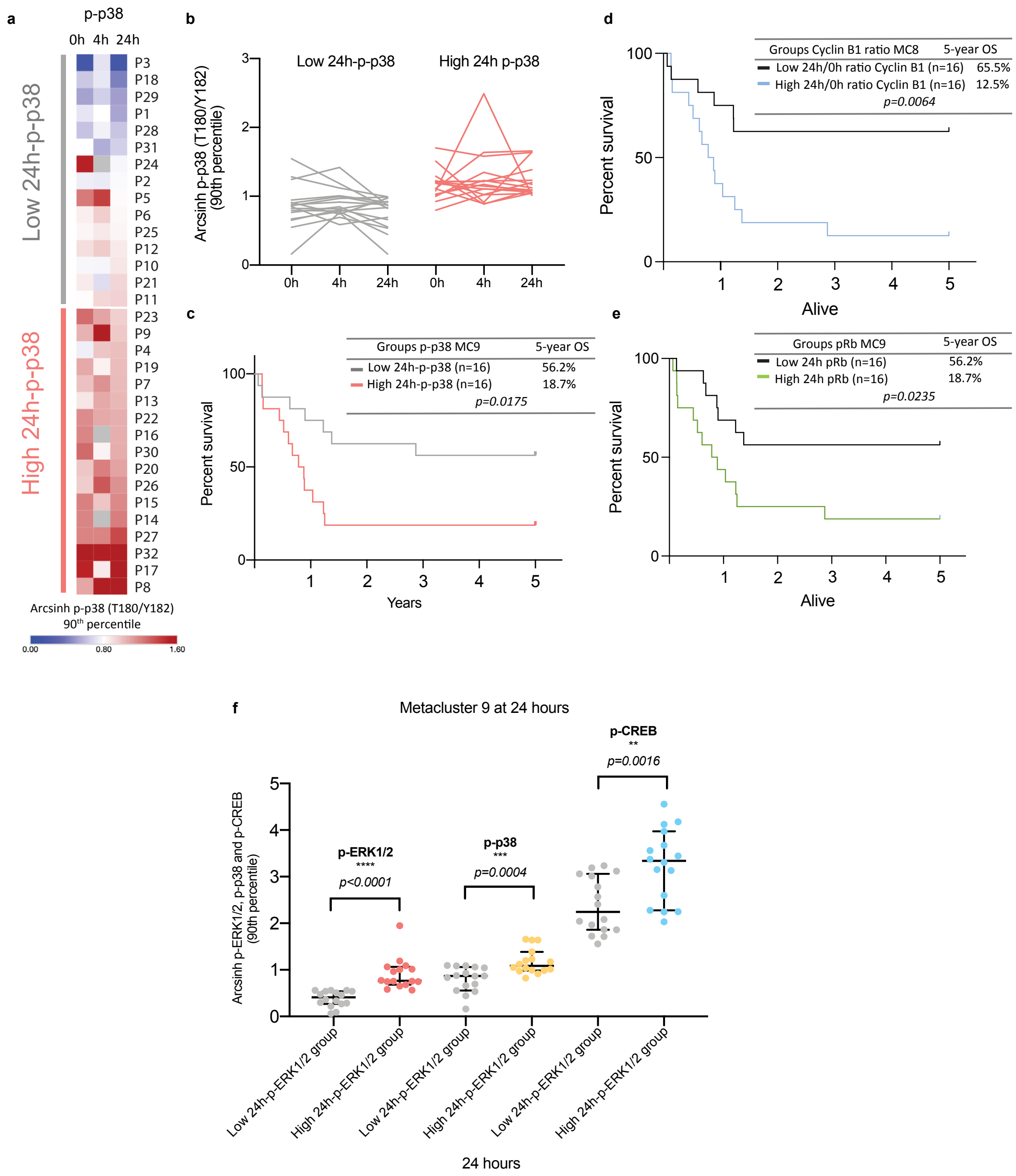

Supplementary Figure 7. p-p38 and p-CREB in MC9

a, Heatmap of the arcsinh transformed 90th percentile p-p38 in MC9 at all timepoints sorted by the 24 h value, divided by median into high and low 24h-p-p38 group. b, Line-graph shows the arcsinh transformed 90th percentile p-p38 value at all timepoint in the two groups. c, Cox LASSO regression analysis identified p-p38 as the second-best predictor of patient two - (HR 3.50) and five (HR 3.39) year survival. Kaplan-Meier curve shows the survival in the 24h sorted high and low p-p38 groups with 16 patients in each group (Log-rank (Mantel-Cox) test p=0.0175, Hazard Ratio (logrank) low/high group 0.3522 and 95% CI of ratio 0.1437 to 0.8631).

d, Cox LASSO regression analysis also identified the ratio (24h/0h) of Cyclin B1 in MC8 (NK cells) to be a predictor of patient 5-year survival The Kaplan-Meier curve shows the survival in the 24h/0h ratio stratified high and low Cyclin B1 groups with 16 patients in each group (Log-rank (Mantel-Cox) test p=0.064. e, Cox LASSO regression analysis also identified the 24h value of pRb in MC9 to be a predictor of patient 5 year survival The Kaplan-Meier curve shows the survival in the 24h stratified high and low Cyclin B1 groups (Log-rank (Mantel-Cox) test p=0.0235. f, Arcsinh transformed 90th percentile values of p-ERK1/2, p-p38, and p-CREB in the high and low 24h-p-ERK1/2 group defined by

the p-ERK1/2 24h median value as shown in the heatmap of figure 2b (n=16 in high group and n=16 in low group). Statistical analysis was done by unpaired tow sided t-tests. 95% CI for pERK1/2 = 0.33 to 0.72, for p-p38 = 0.18 to 0.57, and for p-CREB= 0.35 to 1.35. Exact p-value for pERK1/2 high vs low group: p=1.28 e-5.

Supplementary Figure 8

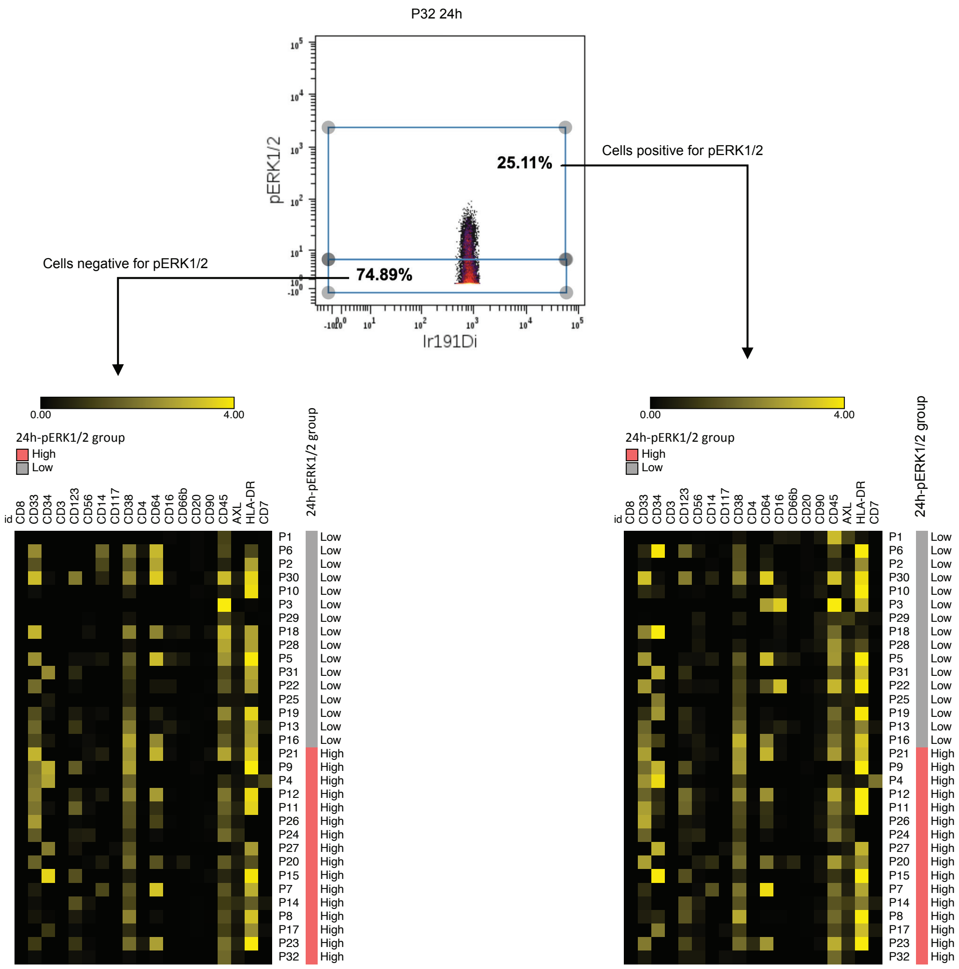

Supplementary Figure 8. Manual gating of pERK1/2 positive and negative cells in MC9. MC9 for all patients were exported and manually gated by bi axial gating. Ir191Di (DNA) on the x-axis, pERK1/2 (T202/Y204) on the y-axis. The gating strategy is shown for patient 32 at 24h after start of induction therapy. The immunophenotype for the pERK1/2 positive cells at 24h for all patients is shown to the right (median marker intensity). Patients are stratified by 24h-pERK1/2 value in MC9. The immunophenotype for the pERK1/2 negative cells at 24h for all patients is shown to the left.

Supplementary Figure 9

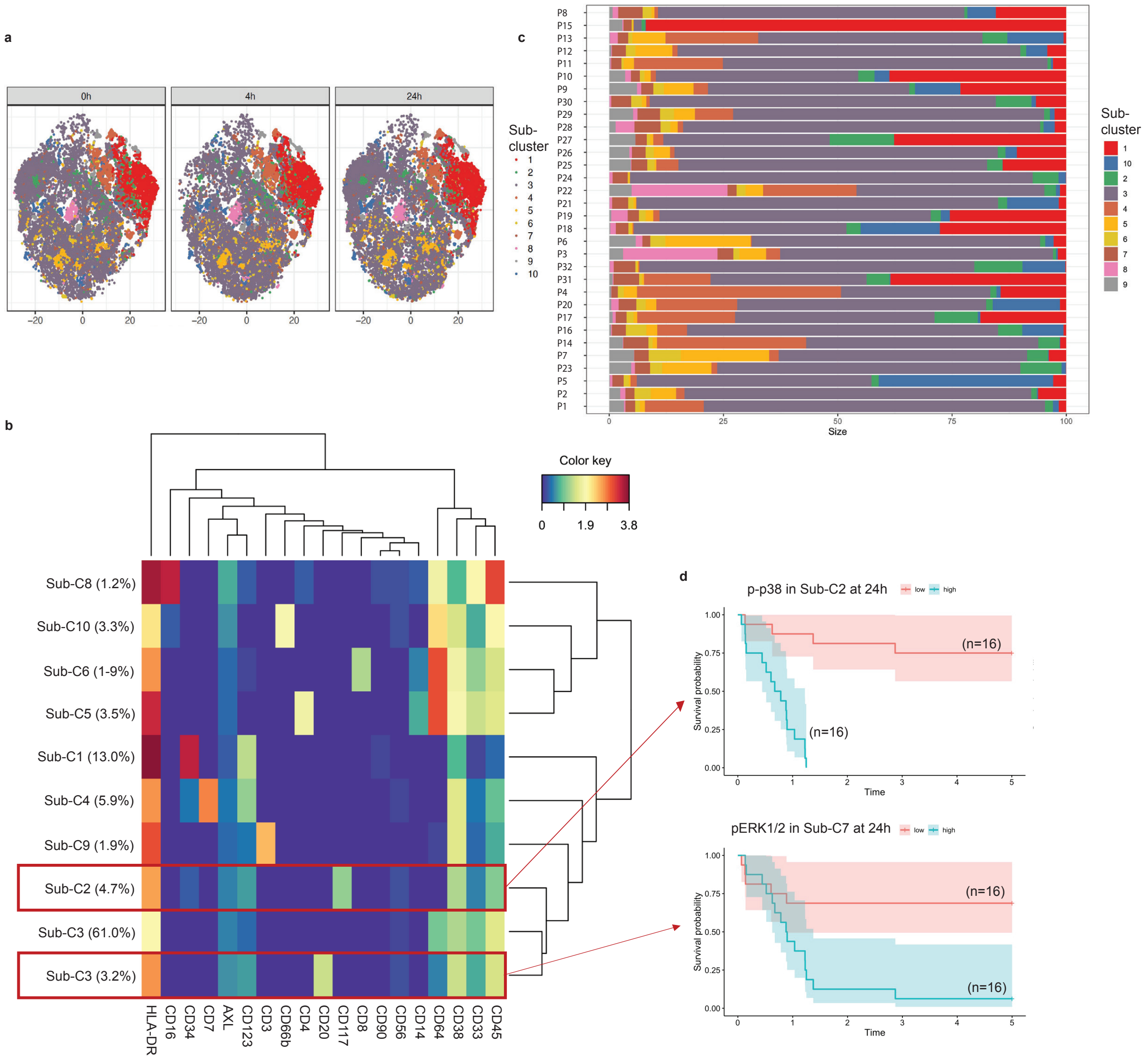

Supplementary Figure 9. New FlowSOM and LASSO Cox regression analysis of MC9. MC9 for all patients was exported and we performed a new FlowSOM analysis of only the cells in MC9. All surface markers were used for clustering, the output was 10 sub-clusters (Sub-Cs). a, t-SNE plots of the 10 Sub-Cs identified within MC9 for all 32 patients at the three different timepoints. b, Heatmap showing the median marker intensity in each of the 10 Sub-Cs for the 32 AML patients at pre-treatment. Size of each Sub-C is shown as percent of MC9. c, stacked bar plot showing the size of each Sub-C for each patient, as percent of MC9. d, a new LASSO cox regression analysis identified the prognostic pERK1/2 and p-p38 signaling in two of the Sub-C (Sub-C2 and Sub-C7) at 24h. Kaplan-Meier curves show the survival when we divide the cohort (n=32) by the median value of p-p38 in Sub-C2 at 24h and pERK1/2 in SubC7 at 24h, respectively, with 16 patients in each group (Error bands show the 95% Confidence interval).

Supplementary Figure 10.

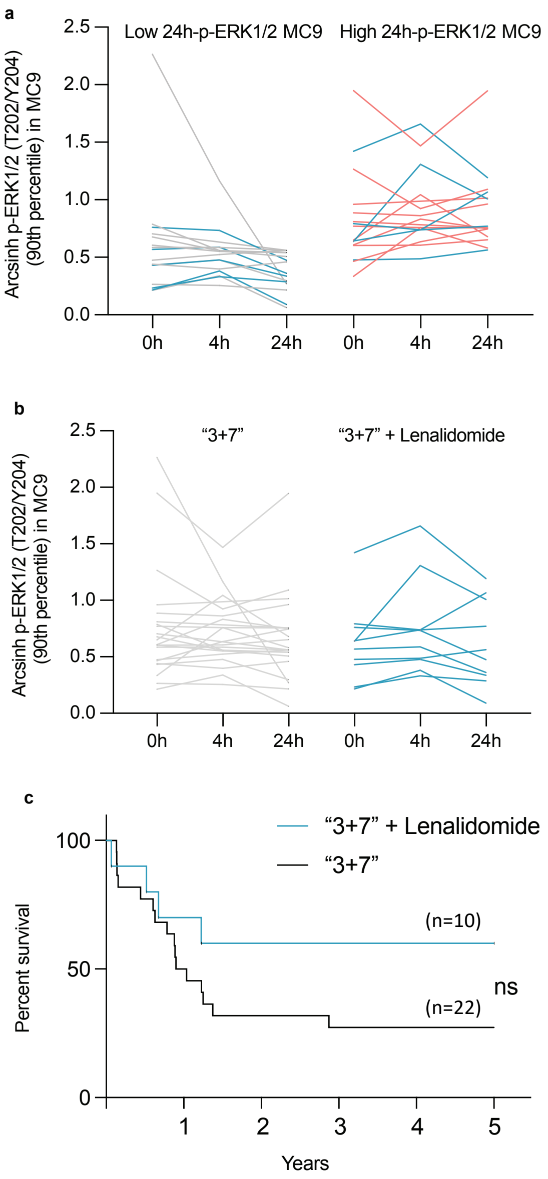

Supplementary Figure 10. Lenalidomide treatment and pERK1/2 signaling in MC9. Ten patients were included in the HOVON 132 study and given a per-oral treatment of Lenalidomide in addition to the induction therapy. a, Line plot of the arcsinh transformed pERK1/2 90th percentile value in MC9 per patient. Patients treated with lenalidomide are colored in blue. There were 5 patients in high 24h-pERK1/2 group and 5 patients in low 24h-pERK1/2 group. b, Line plot showing the pERK1/2 signaling in MC9 for patients receiving standard induction therapy "7+3" and the ten patients who received "7+3" + Lenalidomide. c, Kaplan-Meier curve (n=32) of the 10 patients receiving "7+3" + Lenalidomide and the 22 patients who received only "7+3". No significance in survival between the two groups was found (Log-rank (Mantel-Cox) test (p=0.15) and Gehan-Breslow-Wilcoxon test (p=0.25)).

Supplementary Figure 11

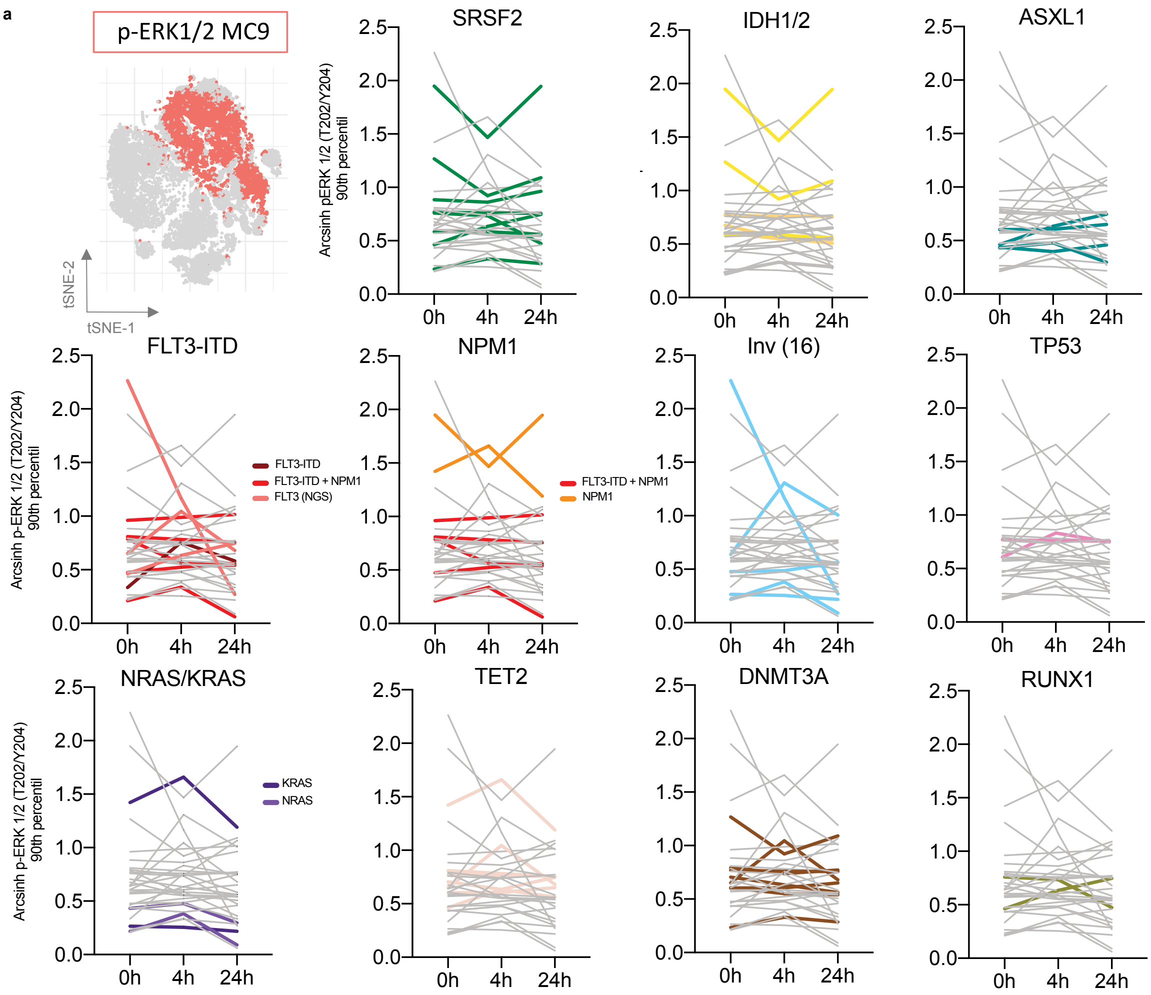

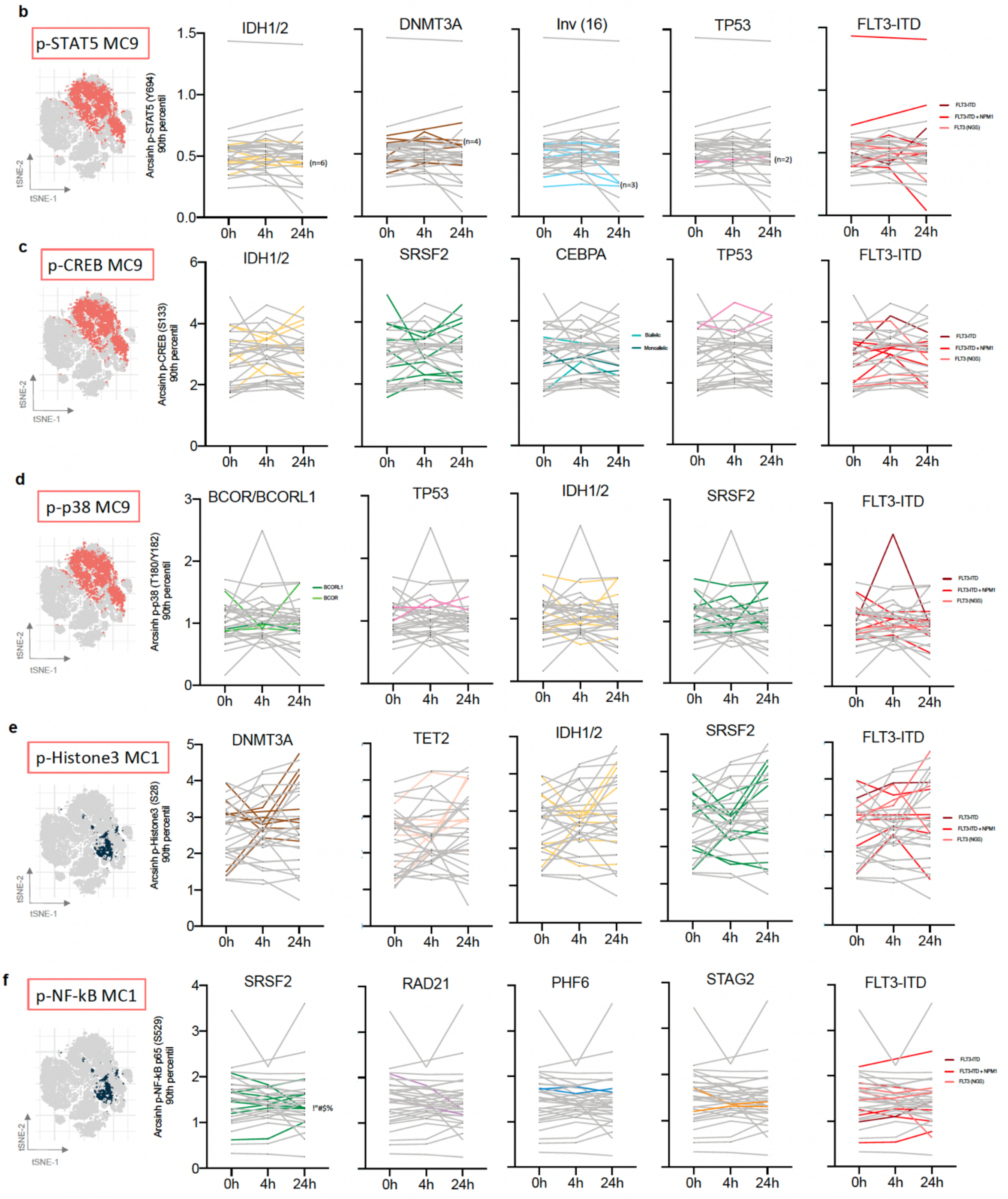

Supplementary Figure 11. Mutations and signaling patterns in MC9 and MC1

a, t-SNE plot visualization of MC9 distribution in the 32 AML patients at pre-treatment, MC9 is highlighted in red, the other metaclusters are shown in grey. The line plots show the arcsinh transformed 90th percentile p-ERK1/2 at all timepoints in MC9 for all the 32 patients in the study. Patients with annotated mutations detected by the Illumina TruSight Myeloid Gene Panel or diagnostic cytogenetics are shown by colored lines. Patients marked by gray lines do not have the annotated mutations or cytogenetic alterations. b, Arcsinh transformed 90th percentile of p-STAT5 in MC9. c, Arcsinh transformed 90th percentile of p-CREB in MC9. d, Arcsinh transformed 90th percentile of p-p38 in MC9. e, Arcsinh transformed 90th percentile of p-Histone3 in MC1. f, Arcsinh transformed 90th percentile of p-NFkB in MC1.

Supplementary Figure 12

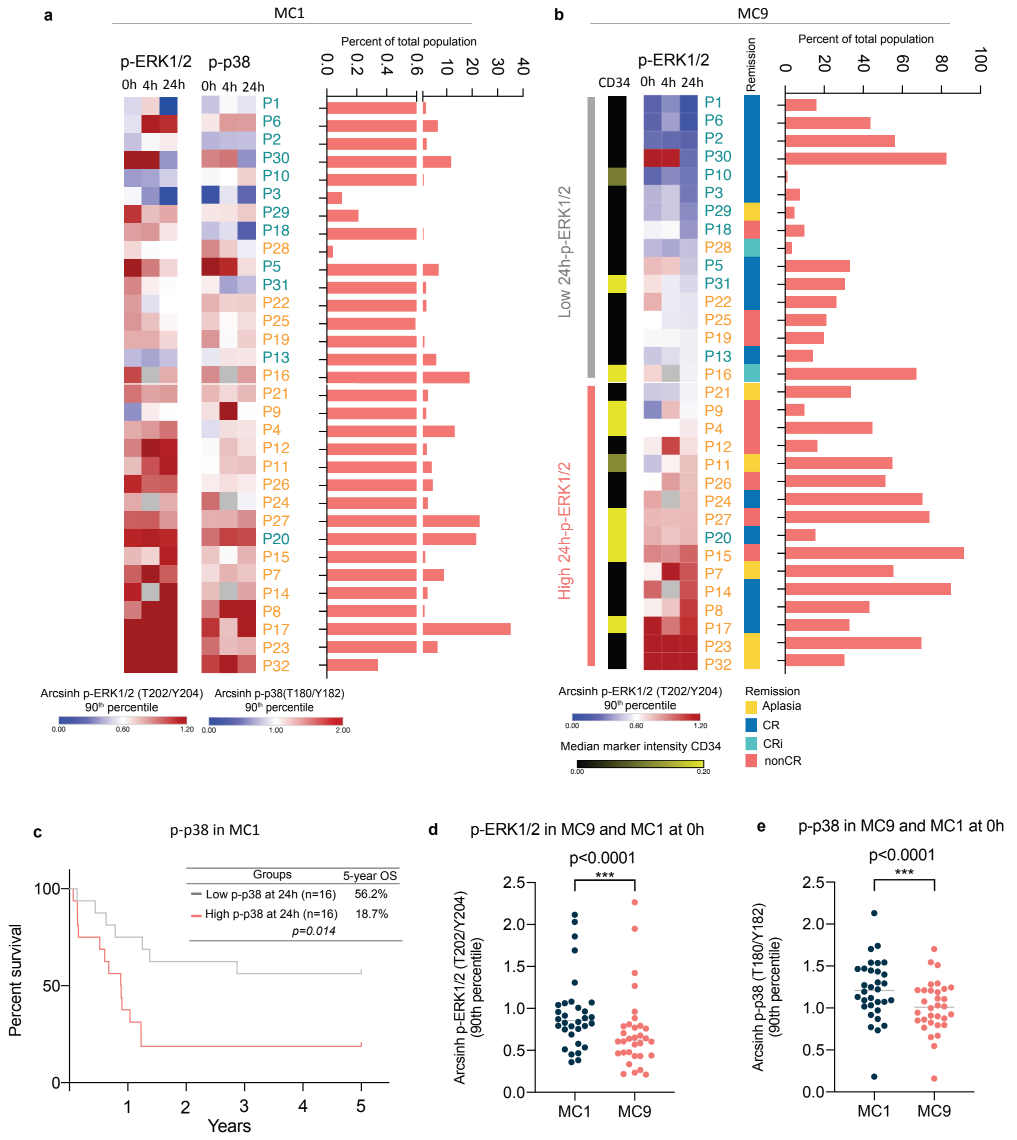

Supplementary Figure 12. p-ERK and p-p38 in MC1 (CD34+ blast cells)

a, Heatmap of the arcsinh transformed 90th percentile of p-ERK1/2 and p-p38 in metacluster (MC) 1. The horizontal bar plot shows the size of MC1 in each patient as percent of total cell population. Patients are sorted by the 24h p-ERK1/2 value in MC9. Patient numbers are color coded by 5-year survival (blue=alive, orange= deceased). b, Heatmap of the arcsinh transformed 90th percentile of p-ERK1/2 in MC9, sorted by the 24-hour value, divided into high and low group by the median 24-hour value. CD34 median marker intensity in MC9 is shown in the heatmap to the left. The horizontal bar plot shows the size of MC9 in each patient as percent of total cell population. c, Kaplan-Meier survival curve (n=32) of the high (n=16) and low (n=16) groups defined by the 24-hour median value for p-p38 in MC1, with 16 patients in each group. p-values are calculated by Log-rank (Mantel-Cox) test for comparison of the survival curves. d, The level of arcsinh transformed 90th percentile p-ERK1/2 in MC1 is significantly higher than in MC9 ($p<0.0001$, exact p-value =1.67e-6) for the 32 patients in our study. e, The level of arcsinh transformed 90th percentile p-p38 in MC1 is significantly higher than in MC9 ($p<0.0001$, exact p-value= 2.04e-5) for the 32 patients in our study. Statistical analysis for d and e was performed by a two-sided paired t-tests.

Supplementary Figure 13

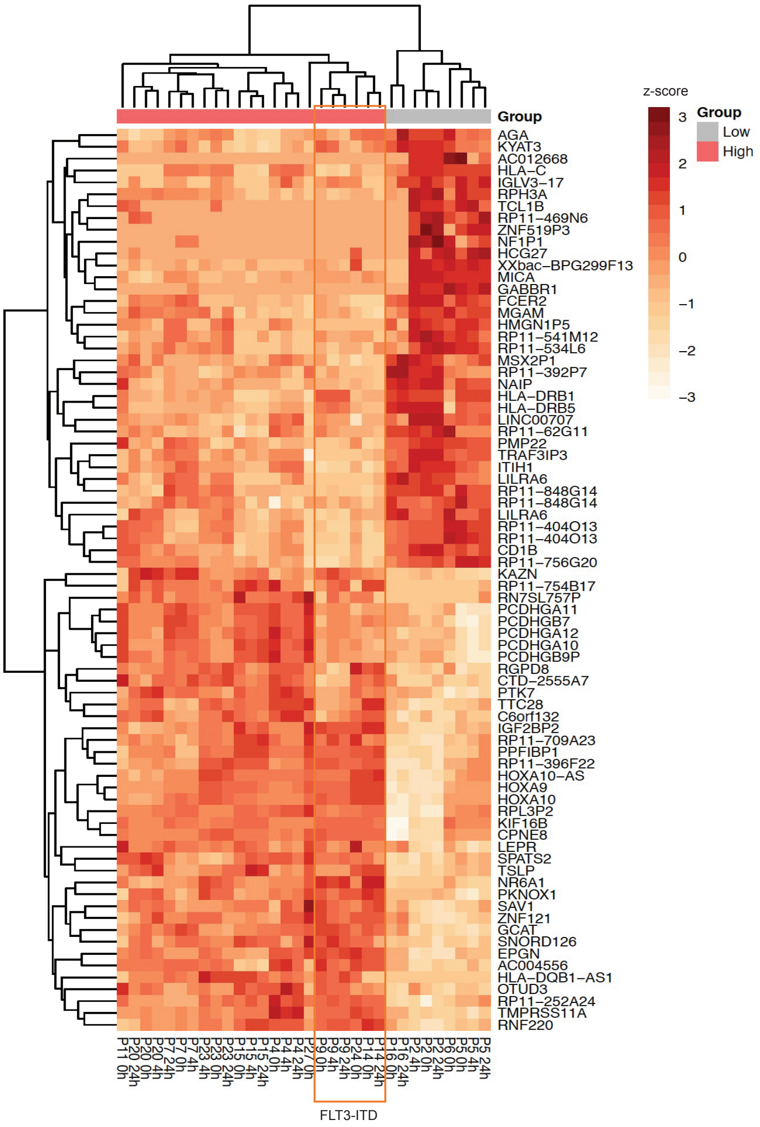

Supplementary Figure 13. RNAseq bulk analysis. A student's t-test with FDR cut-off<0.05 was performed between low vs high 24h-pERK1/2 groups at all time points for the 14 patients in our cohort with RNAseq data. All genes in our dataset were included in this analysis (n=50.668) and 76 significant genes were identified. The significant genes were clustered by unsupervised hierarchical clustering (Euclidean distance) as shown in the heatmap. Patients in low and high 24h-pERK1/2 groups clustered separately. The three patients with FLT3-ITD clustered together within the 24h-pERK1/2 group.

Supplementary Figure 14

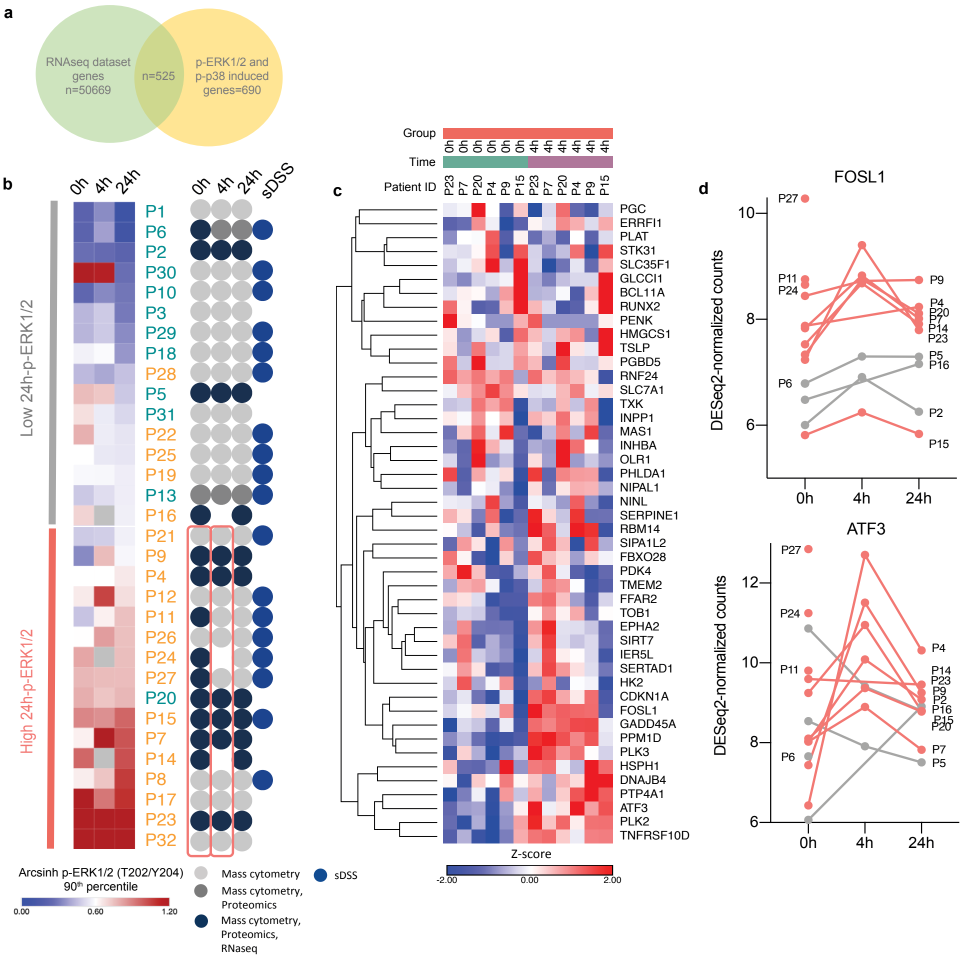

Supplementary Figure 14. RNAseq 0h and 4 hours in high 24h-p-ERK1/2 group

a, Venn diagram showing that 525 p-ERK1/2 and p-p38 induced genes were identified among our RNAseq data. b, Heatmap of the 90th percentile arcsinh transformed p-ERK1/2 in M9 mass cytometry data. The high and low groups used for RNAseq data analysis were defined by the 24-hour p-ERK1/2 median value in MC9. Patient survival and an overview of the patient sample material and the analysis performed is shown to the right as in Figure 4a (sDSS= selective drug sensitivity score). The groups used for statistical testing are annotated in red squares (24h-pERK1/2 high group, 0h vs 4h samples) c, Hierarchical clustering (Euclidean distance) of the differentially expressed genes identified by a paired, two-sided students t-test between the pre-treatment sample (0h) and 4 hours sample in the 6 patients in 24h p-ERK1/2 high group with 4-hour sample. Adjustment for multiple comparisons was not used when identifying these genes. Patients are sorted by sampling timepoint; 0h and 4 hours. Among the 525 p-ERK1/2 and p-p38 induced genes there were 46 differentially expressed genes, only genes with p-values <0.05 are shown. The exact p-values for each gene are shown in the source data of this figure. The heatmap shows z-scored DESeq2 normalized counts. d, Line plots showing the DESeq2 normalized counts for each patient at the three different time points for FOSL1 and ATF3. Patients in 24h p-ERK1/2 low group are colored in grey, and patients in 24h p-ERK1/2 high group in red.

Supplementary Figure 15

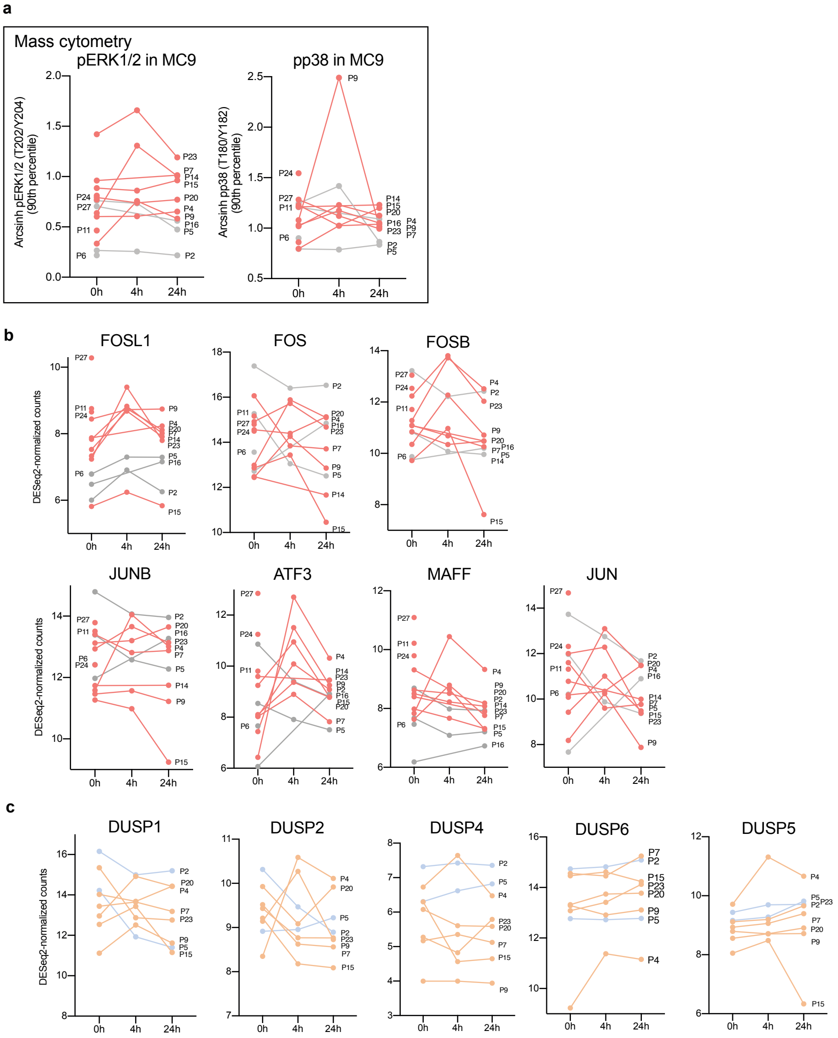

Supplementary Figure 15. AP-1 transcription factor genes and DUSPs

a, Arcsinh Transformed 90th percentile mass cytometry data on p-ERK1/2 and p-p38 at the three sampled timepoints in MC9 for the 14 patients that was analyzed by RNAseq. b, DESeq2 normalized counts for the 14 patients analyzed by RNA sequencing shown for different members of the AP-1 transcription factor family. Patients in 24h p-ERK1/2 low group are colored in grey, patients in 24h p-ERK1/2 high group in red. c, DESeq2 normalized counts for the 14 patients analyzed by RNA sequencing shown for different members of the DUSP family. Patients in 24h p-ERK1/2 low group are colored in blue, patients in 24h pERK1/2 high group in orange.

Supplementary Figure 16

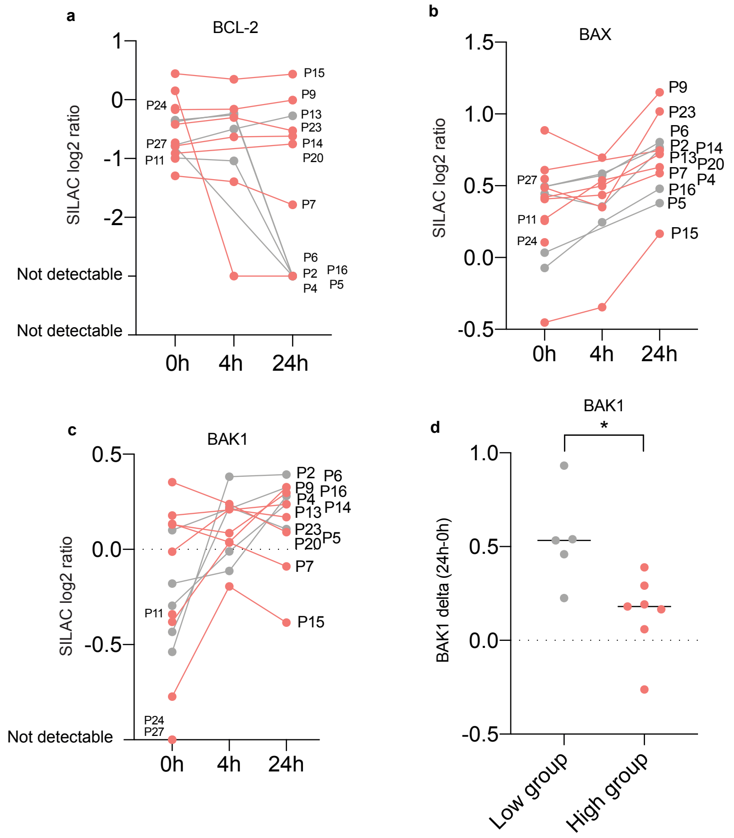

Supplementary Figure 16. Anti-apoptotic BCL-2 decrease at 24 hours in patients responding to induction therapy

a, SILAC log2 ratio (Light/heavy) of the anti -apoptotic BCL-2 (n=15). SILAC log2 ratio (Light/Heavy) of BCL-2 was decreased to a non-detectable level for patient 4 at 4-hours and the four patients in low group (P6, P2, P16 and P5) at 24-hours. b, SILAC log2 ratio (Light/heavy) of the pro-apoptotic protein BAX in all patients analyzed by proteomics (n=15) at the three different sampling timepoints. c, SILAC log2 ratio (Light/heavy) of the pro-apoptotic protein BAK1 in all patients analyzed by proteomics (n=15) at the three different sampling timepoints. BAK1 was not detected in patient 24 and 27 at pre-treatment. d, The change in protein expression (SILAC log2 ratio (Light/Heavy)) of BAK1 from pre-treatment to 24 hours was calculated and compared between the 24h- p-ERK1/2 low and high groups. Patients in low group (n=5) had a significantly higher increase in the pro-apoptotic BAK1 protein compared to the high group (n=7) (Unpaired t-test, with two tailed p-value 0.0145). High and low group is defined by the 24h-p-ERK1/2 median value in MC9 as shown in Fig. 3b. Patients in 24h-p-ERK1/2 high group are colored in red, patients in low group in grey.

and b,

Supplementary Figure 17

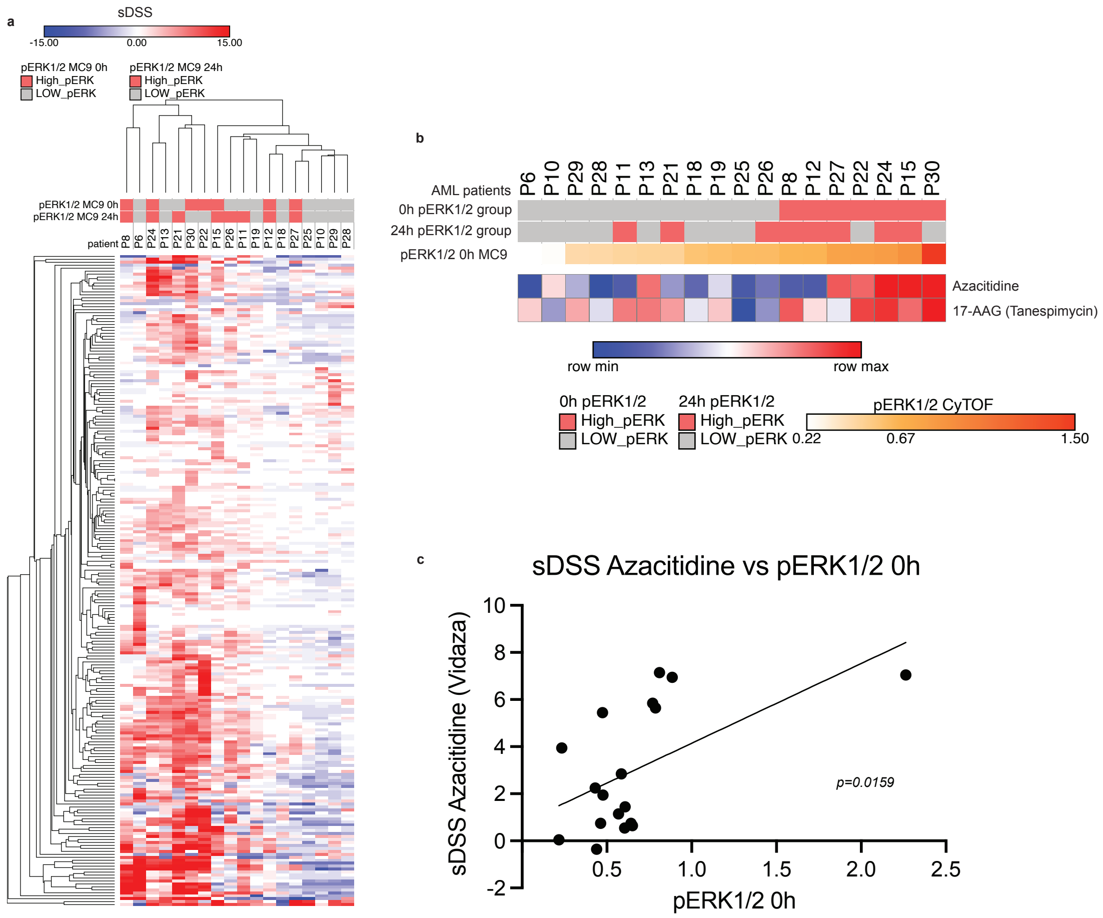

Supplementary Figure 17. Drug sensitivity and resistance testing (DSRT) show that patients in high 24h-pERK1/2 group are more sensitive to azacytidine. a, Heatmap of the DSRT data shown as selective drug sensitivity score (sDSS). Only drugs that had sDSS values under or over 5.0 for at least one patient were included in the analysis. pERK1/2 group at 24h and 0h for each patient is shown in red and grey at the top of the heatmap. b, A two-sided students t-test between low and high 0h p-ERK1/2 group (as annotated in the top color-coded bar in heatmap a and b) identified azacytidine (*p=0.0128*) and tanespimycin (*p=0.0225*) as the two most efficient drugs in patients with high pERK1/2 at pre-treatment. c, Simple linear regression of pERK1/2 at 0h in MC9 vs sDSS for azacytidine for the 18 patients with DSRT data in our cohort show the significant correlation (*p=0.0159*).

Supplementary Figure 18

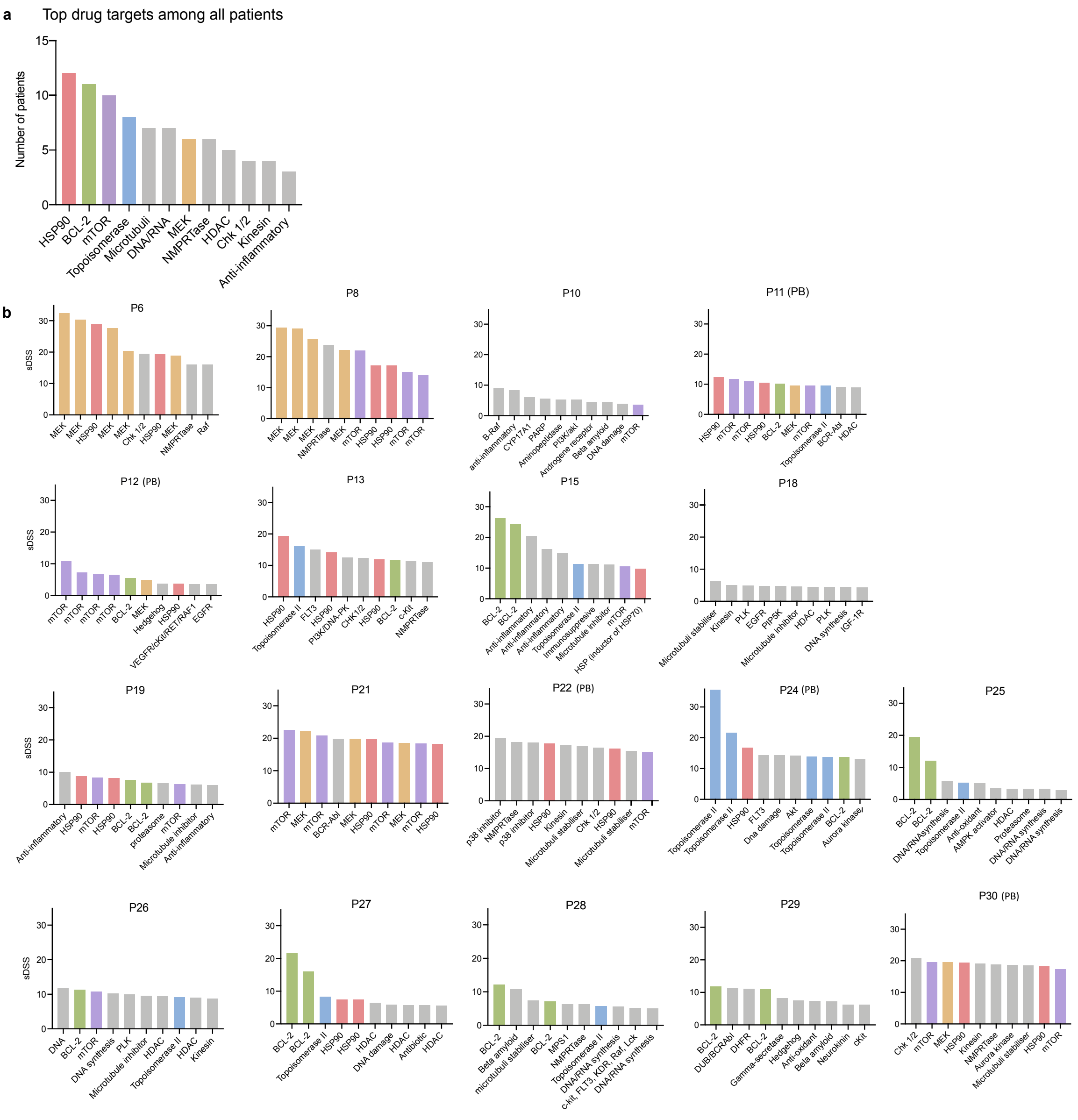

Supplementary Figure 18. Top 10 drug targets by sDSS per patient

a, The 10 drugs with the highest selective DSS (sDSS) for each patient was selected and the most common drug targets among all patients (n=18) was summarized. The four top drugs and MEK inhibitors are annotated by color. b, sDSS for the top 10 drugs for each patient. Top drugs and MEK inhibitors are colored as in figure a. Drug sensitivity and resistance testing (DSRT) was performed on peripheral blood from patients marked with (PB). All the others were bone marrow samples.

Supplementary Figure 19

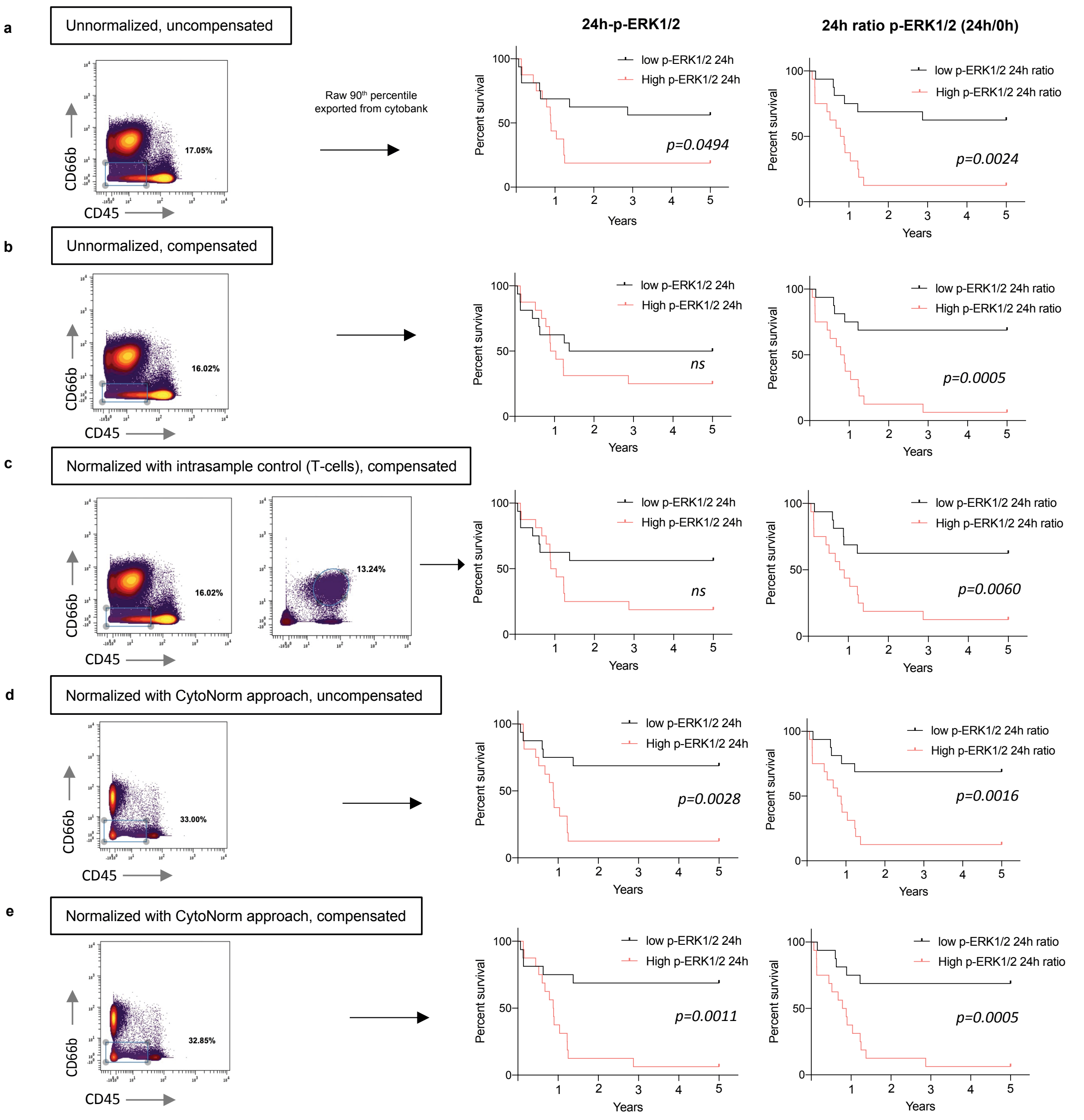

Supplementary Figure 19. CytoNorm normalization and Catalyst compensation, manual gating of CD45low, CD66low cells.

Manual, bi-axial gating of unnormalized, uncompensated, normalized and compensated 90th percentile raw data of p-ERK 1/2 in the CD45low, CD66low cell population (exported from cytobank) for the 32 patients in our cohort. Contour scatter plots to the left (colored by density) of AML patient 1 (P1) pre-treatment sample, show the gating strategy of CD45low, CD66low cells. The CytoNorm approach was used for normalization, the Catalyst approach was used for compensation. Median values of 24h-p-ERK1/2 and 24h ratio p-ERK1/2 (24h/0h) divided patients into two groups, with 16 patients in each group, and 5-year survival Kaplan Meier curves show the difference between the two groups. p-values were calculated by Log-rank (Mantel-cox) test. a, Original, unnormalized, uncompensated data. b, Unnormalized, compensated data. c, pERK1/2 in the CD45low, CD66low population was divided by the pERK1/2 level of CD4+ T cells in the same sample for normalization. d, Normalized, uncompensated data. e, Normalized, compensated data.

Supplementary Figure 20

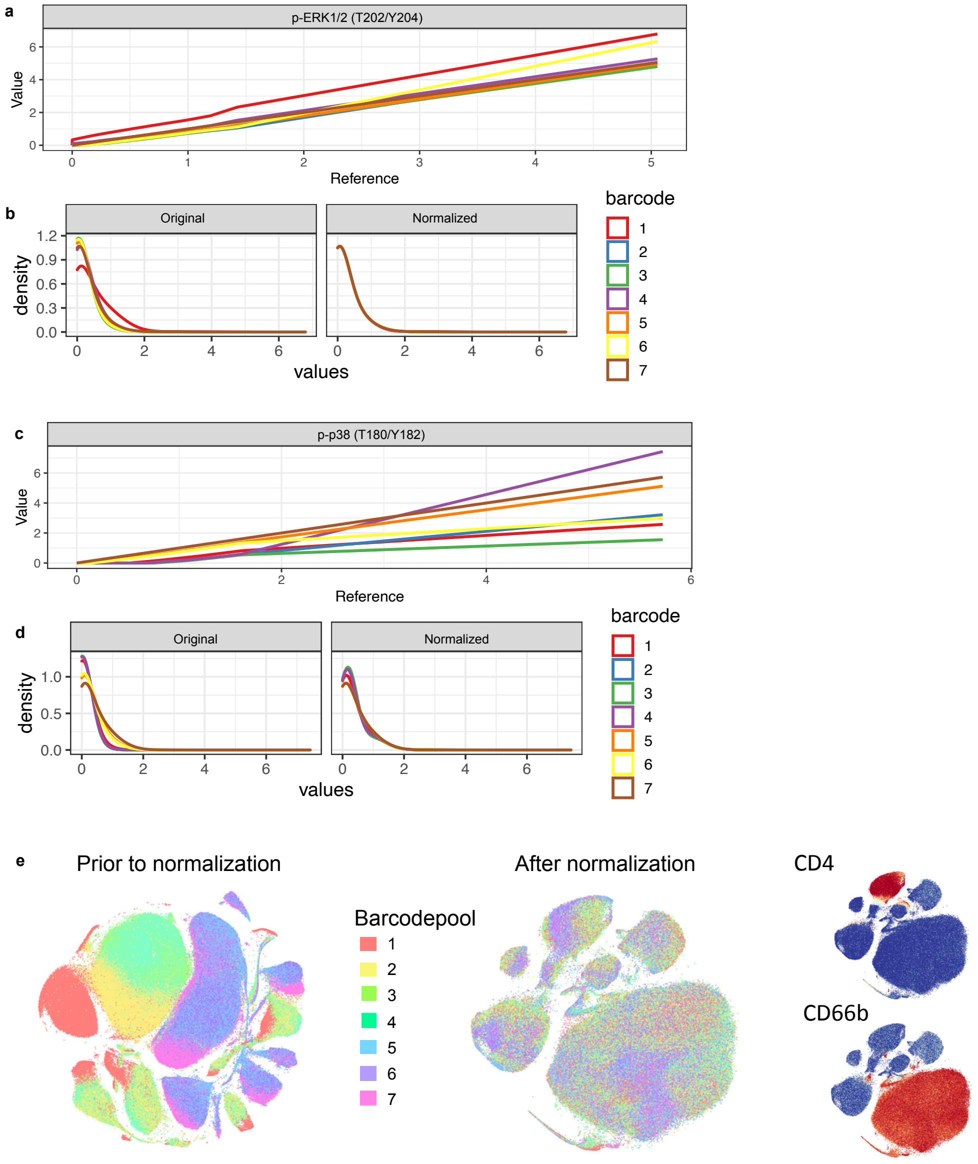

Supplementary Figure 20. Prior to and after CytoNorm normalization

Normalization was performed on all markers (both intracellular and surface markers), the original versus normalized data for p-ERK1/2 and p-38 are visualized in this figure. a, Original values of p-ERK1/2 in the reference samples in barcodepool 1-7. b, Distribution of the reference sample in barcodepool 1-7 before (original) and after (Normalized) normalization with the CytoNorm approach, normalized to the reference sample in barcodepool 7. c, Original values of p-p-38 in the different reference samples in barcodepool 1-7. d, Distribution of the reference sample in barcodepool 1-7 before (original) and after (Normalized) normalization with the CytoNorm approach, normalized to the reference sample in barcodepool 7. e, t-SNE plots showing the 7 reference samples (mix of peripheral blood from 7 healthy donors) in the 7 barcodepools (batches) prior to normalization and after normalization the right. CD4 and CD66b staining is shown in the normalized samples.

Supplementary Figure 21

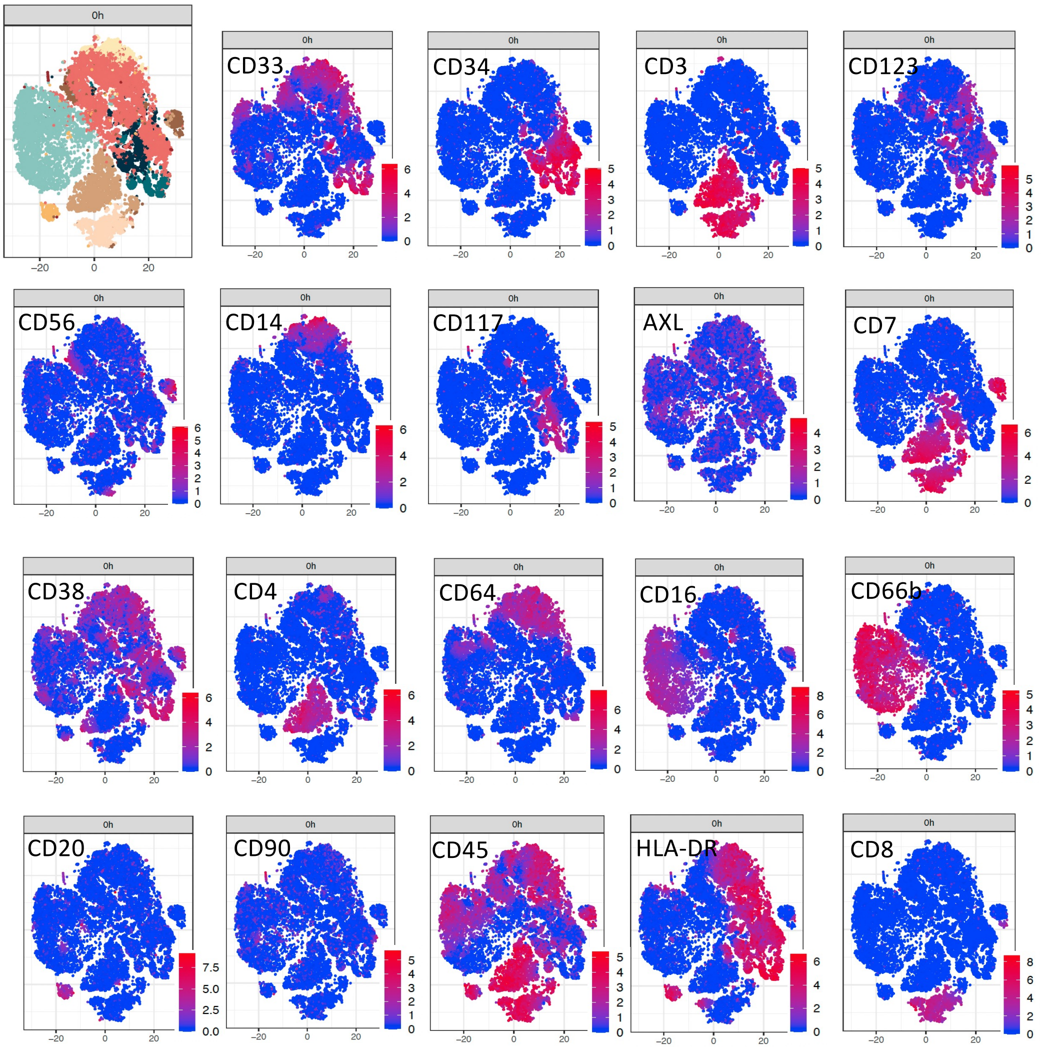

Supplementary Figure 21. Expression of surface markers in all metaclusters identifyed by FlowSOM. t-SNE plots showing the single cell expression level (arcsinh transformed dual counts) of all surface markers in our antibody panel (except for CD25 and CD11b). A color coded overlay t-SNE plot showing the different metalcusters identified among the 32 patients at pre-treatment is shown in the upper left, annotation as in figure 2.

Supplementary Figure 22

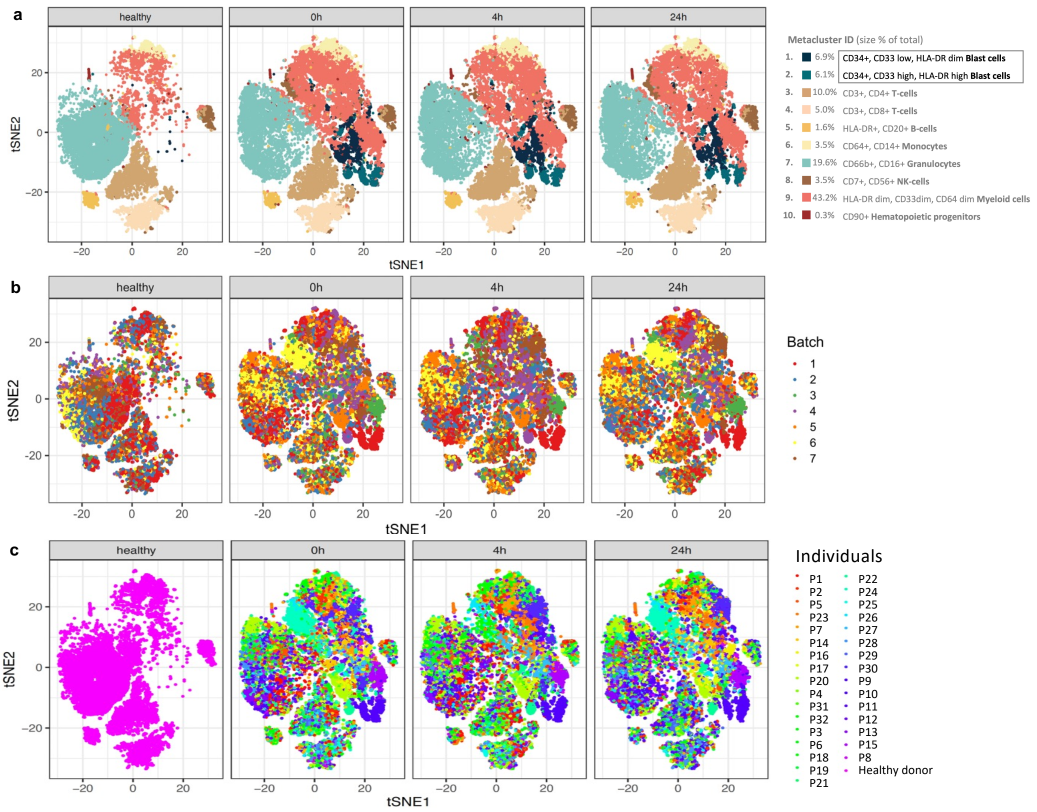

Supplementary Figure 22. Distribution of barcodepools (batches) and patient ID in metaclusters identified by FlowSOM. a, t-SNE overlay of the 10 different metaclusters, metacluster ID is shown to the right. b, t-SNE color-coded overlay of the 7 different batches (barcodepools) to be able to assess for potential batch effects. c, t-SNE color-coded overlay of the 32 AML patients (three timepoints) and the healthy donors (pink).

Supplementary Figure 23

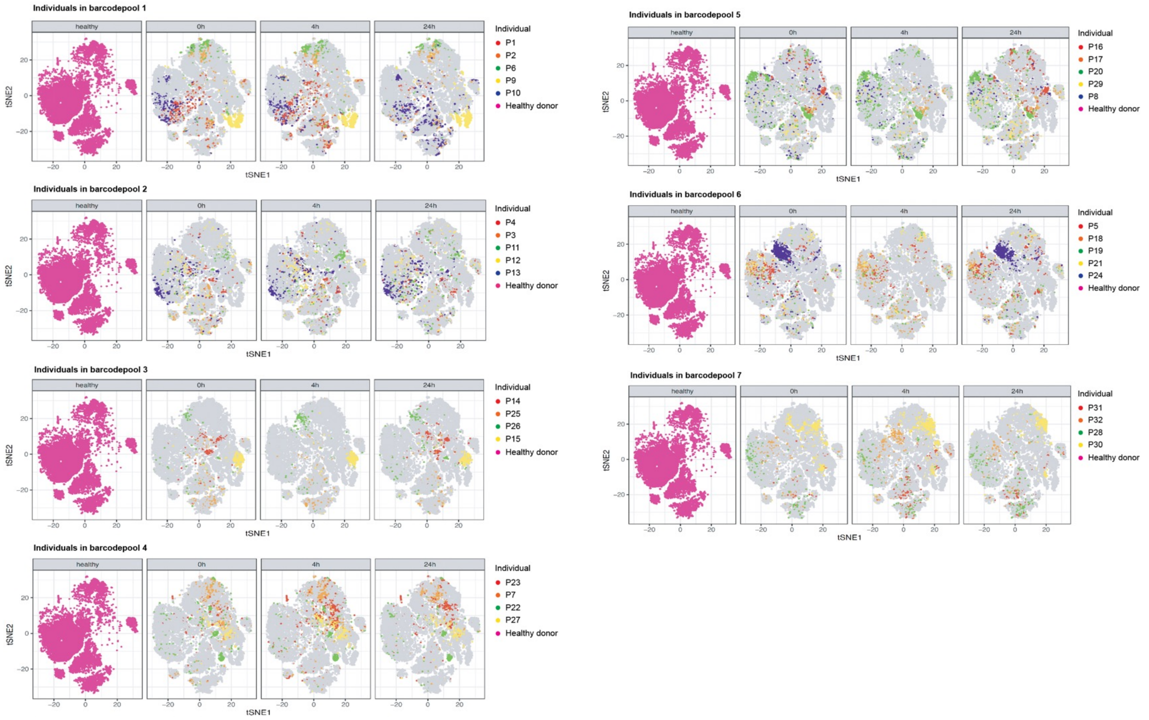

Supplementary Figure 23. Distribution of patient ID in each barcodepool (batch) in metaclusters identified by FlowSOM t-SNE color-coded overlay of the 32 AML patients at all timepoints and 7 healthy donors (pink) in each barcodepool (batch).

Supplementary Figure 24

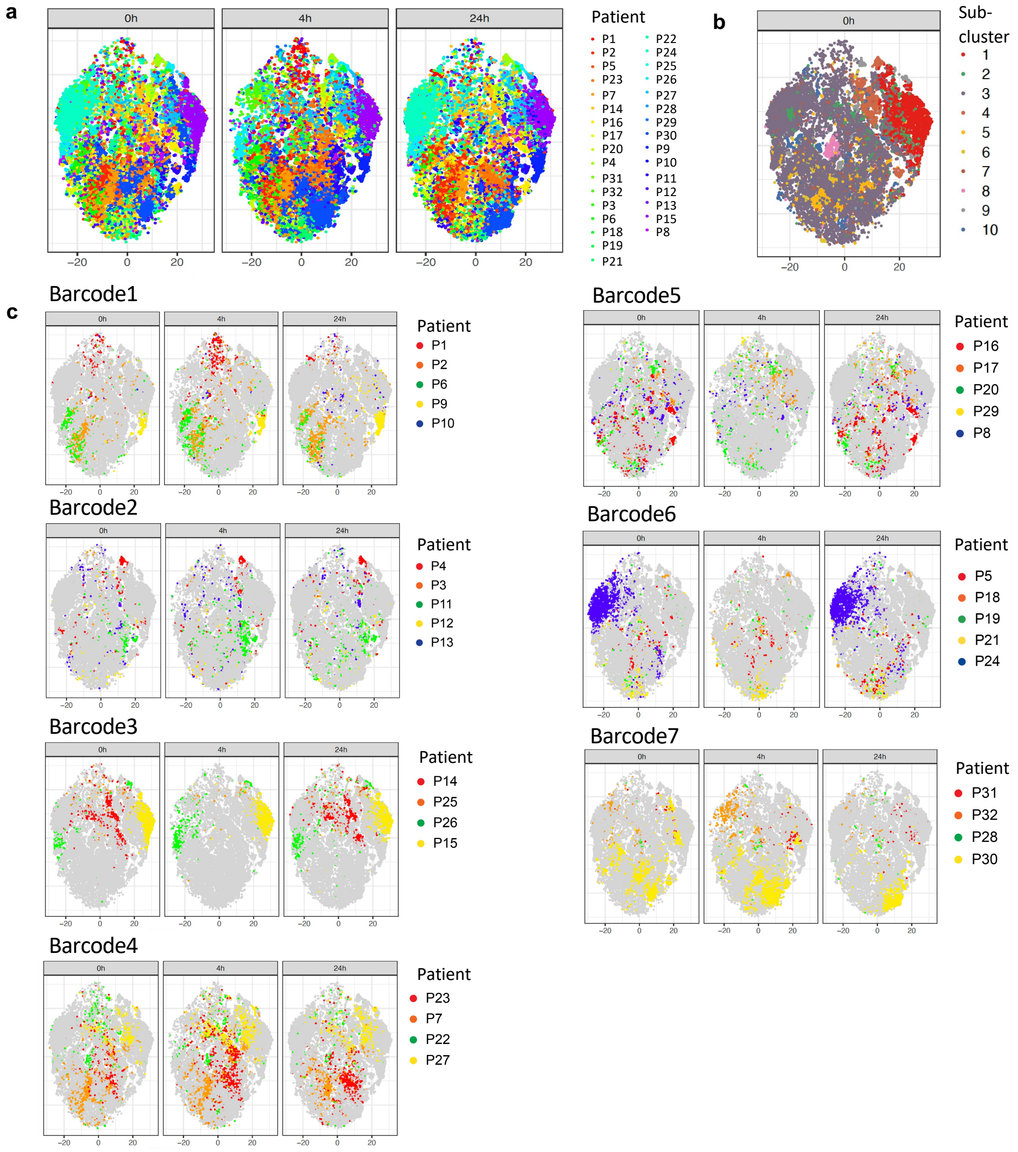

Supplementary Figure 24. Distribution of patient ID in each barcodepool (batch) in sub-clusters identified by FlowSOM in MC9.a, t-SNE color coded overlay of the 32 AML patients at all timepoints. b, t-SNE color-coded overlay of the different sub-clusters identified within MC9. c, t-SNE color coded overlay of patient ID in the 7 different barcodepools.

Supplementary Figure 25

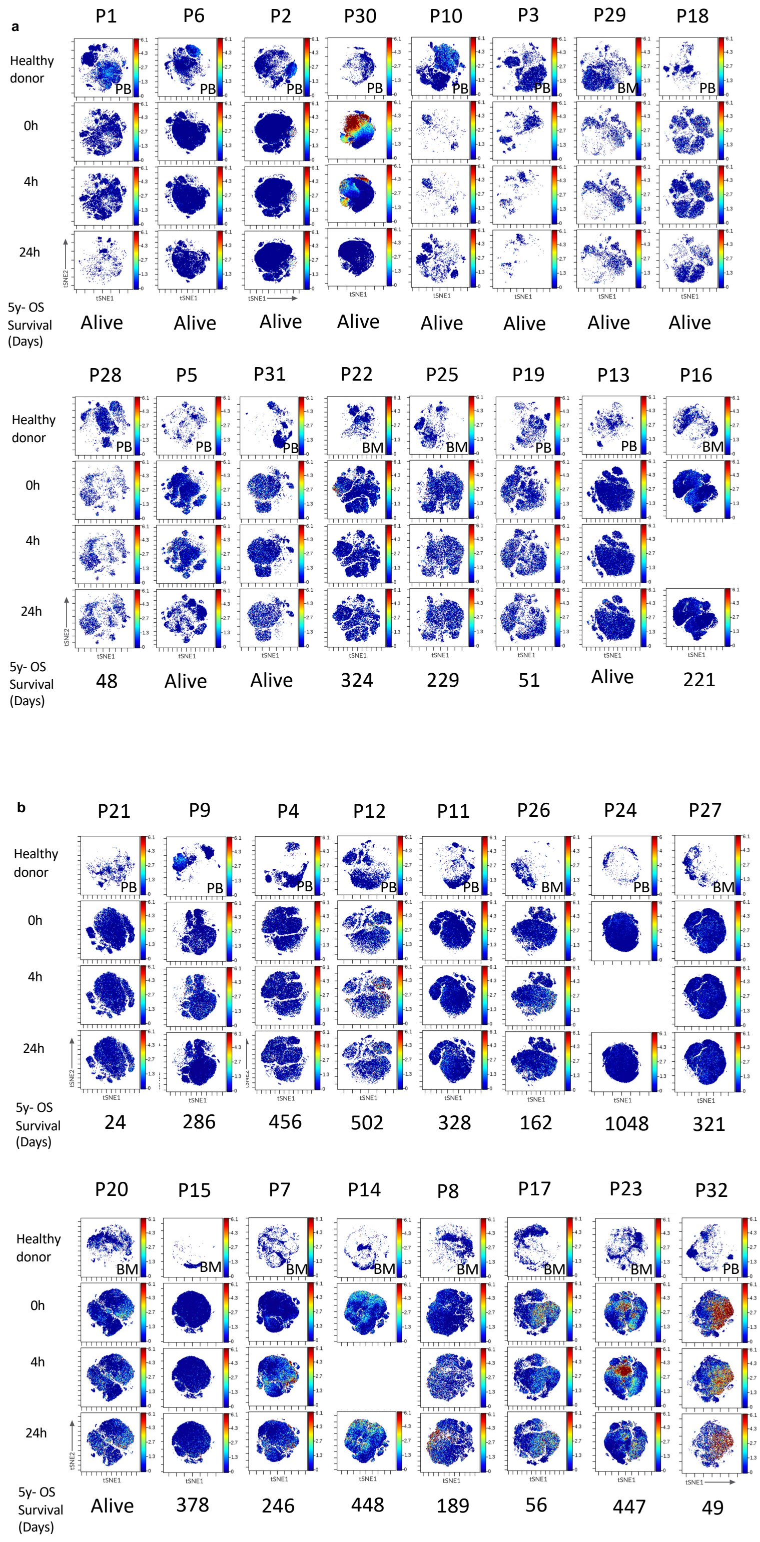

Supplementary Figure 25. pERK1/2 staining in MC9. All cells in metacluster (MC) 9 for each patient were exported and a new viSNE analysis of all cells including the healthy donor included in the same barcodepool (batch) as the patient was performed (Clustered on surface markers). The healthy donor was bone marrow (BM) or peripheral blood (PB), which is specified in the plots. a, t-SNE plots showing the single-cell expression of pERK1/2 (Raw dual counts) in 24h-low pERK1/2 group. Patients (P) are sorted by pERK1/2 24 value in MC9 from low

to high. Patient 5-year overall survival is shown in days below each patient. b, t-SNE plots showing the single-cell expression of pERK1/2 (Raw dual counts) in 24h-high pERK1/2 group.
