# Supplementary material for: Early response evaluation by single cell signaling profiling in acute myeloid leukemia
Source: Nat Commun. 2023 Jan 7;14:115. doi: 10.1038/s41467-022-35624-4 (PMC9825407; doi:10.1038/s41467-022-35624-4)
Supplement: Supplementary file 3 — Description of Additional Supplementary Files [file 41467_2022_35624_MOESM3_ESM.pdf]

Description of Additional Supplementary Files

File Name: Supplementary Data 1
Description: Proteomics output

File Name: Supplementary Data 2
Description: Patient characteristics

File Name: Supplementary Data 3
Description: Antibody panel

File Name: Supplementary Data 4
Description: pERK and p38 inducible genes

File Name: Supplementary Data 5
Description: Mutations (TruSight myeloid panel) and cytogenetics

File Name: Supplementary Data 6
Description: Selective drug sensitivity score
