# Supplementary material for: Early response evaluation by single cell signaling profiling in acute myeloid leukemia
Source: Nat Commun. 2023 Jan 7;14:115. doi: 10.1038/s41467-022-35624-4 (PMC9825407; doi:10.1038/s41467-022-35624-4)
Supplement: Supplementary file 10 — Reporting Summary [file 41467_2022_35624_MOESM10_ESM.pdf]

nature portfolio

Corresponding author(s): Bjørn Tore Gjertsen Last

Last updated by author(s): 2022. 12. 09

Reporting Summary

Nature Portfolio wishes to improve the reproducibility of the work that we publish. This form provides structure for consistency and transparency in reporting. For further information on Nature Portfolio policies, see our Editorial Policies and the Editorial Policy Checklist.

Statistics

For all statistical analyses, confirm that the following items are present in the figure legend, table legend, main text, or Methods section.

n/a	Confirmed	
☐	☒	The exact sample size (*n*) for each experimental group/condition, given as a discrete number and unit of measurement
☐	☒	A statement on whether measurements were taken from distinct samples or whether the same sample was measured repeatedly
☐	☒	The statistical test(s) used AND whether they are one- or two-sided<br>*Only common tests should be described solely by name; describe more complex techniques in the Methods section.*
☐	☒	A description of all covariates tested
☐	☒	A description of any assumptions or corrections, such as tests of normality and adjustment for multiple comparisons
☐	☒	A full description of the statistical parameters including central tendency (e.g. means) or other basic estimates (e.g. regression coefficient) AND variation (e.g. standard deviation) or associated estimates of uncertainty (e.g. confidence intervals)
☐	☒	For null hypothesis testing, the test statistic (e.g. *F*, *t*, *r*) with confidence intervals, effect sizes, degrees of freedom and *P* value noted<br>*Give P values as exact values whenever suitable.*
☒	☐	For Bayesian analysis, information on the choice of priors and Markov chain Monte Carlo settings
☒	☐	For hierarchical and complex designs, identification of the appropriate level for tests and full reporting of outcomes
☒	☐	Estimates of effect sizes (e.g. Cohen's *d*, Pearson's *r*), indicating how they were calculated

Our web collection on statistics for biologists contains articles on many of the points above.

Software and code

Policy information about availability of computer code

Data collection	For proteomics: MaxQuant software version 1.5.2.8, The spectra were searched against the concatenated forward and reversed-decoy Swiss-Prot Homo sapiens database (version 2018_02), using the Andromeda search engine. TruSight myeloid panel analysis: MiSeqReporter version 2.6.2.3 (Illumina) mapping to the human genome reference hg19 and variant calling using Somatic variant caller 3.5.2.1.
Data analysis	R version 4.1.0 (2021-05-18), FlowSOM package in R (FlowSOM version 2.0.0), R package glmnet version 4.1-2. CATALYST (Cytometry Data analysis tools) pipeline. [https://doi.org/10.18129/B9.bioc.CATALYST], CytoNorm pipeline, single cell debarcode algorithm (https://github.com/nolanlab/single-cell-debarcoder), For DSRT data: online tool Breeze (https://breeze.fimm.fi/28489_mc43oty0nzywmcaxnje3nzgwnzaw/index.php#), For proteomics: Perseus version 1.6.1.1. For RNAseq normalization DESEQ2 R package (version 3.15)

For manuscripts utilizing custom algorithms or software that are central to the research but not yet described in published literature, software must be made available to editors and reviewers. We strongly encourage code deposition in a community repository (e.g. GitHub). See the Nature Portfolio guidelines for submitting code & software for further information.

Data

Policy information about availability of data

All manuscripts must include a data availability statement. This statement should provide the following information, where applicable:

- Accession codes, unique identifiers, or web links for publicly available datasets
- A description of any restrictions on data availability
- For clinical datasets or third party data, please ensure that the statement adheres to our policy

The mass cytometry data generated in this study have been deposited in the FlowRepository database under accession code RvFr0LLv9McDJ89jgK50G4lwnfDFRTrcMelxYgnSlcE2Cymrpf2qh2NaWybtWDNH. [http://flowrepository.org/id/RvFr0LLv9McDJ89jgK50G4lwnfDFRTrcMelxYgnSlcE2Cymrpf2qh2NaWybtWDNH.]. The proteomics data generated in this study have been deposited in the ProteomeXchange Consortium via the PRIDE partner database under accession code PXD031916. [https://www.ebi.ac.uk/pride/archive?keyword=PXD031916] Proteomics output data is provided in this manuscript as Supplementary information file 1. The raw fastq files from the RNA sequencing data are deposited in the NCBI gene expression omnibus (GEO) repository and is available under the accession code GSE218664. [https://www.ncbi.nlm.nih.gov/geo/query/acc.cgi?acc=GSE218664] The DSRT data and the results of the NGS (Illumina TruSight myeloid panel) data generated in this study are provided in the Source Data file. Public availability of raw data DNA sequencing data (TruSigh myeloid panel) is not compliant with Norwegian regulations (GDPR) or allowed by the patient consent. For non-commercial academic use, please contact B.T.G. for further information (bjorn.gjertsen@uib.no), which will require an ethical application to the Regional Committee for Medical Research Ethics in Norway (REK). The time the data will be available for the requester will need to be indicated in the application and discussed with REK and further information can be found in the REK website [https://rekportalen.no]. In the proteomics data analysis the spectra were searched against the concatenated forward and reversed-decoy Swiss-Prot Homo sapiens database (version 2018_02), using the Andromeda search engine. The RNA sequencing fastq files were aligned to human genome GRCh38.p13 using HISAT2 aligner. Secondary analysis of the TruSight myeloid data was done using MiSeqReporter version 2.6.2.3 (Illumina) mapping to the human genome reference hg19 and variant calling using Somatic variant caller 3.5.2.1. The remaining data are available within the Article, Supplementary Information or Source Data file. Source data are provided with this paper.

Human research participants

Policy information about studies involving human research participants and Sex and Gender in Research.

Reporting on sex and gender	Patients were consecutively included in this study, therefore there were no selection of patients based on sex in this study. 19 males and 13 females were included in this study.
Population characteristics	AML patients included in this study were younger fit AML patients eligible for standard "7+3" induction therapy. We have to large extent followed the inclusion criteria of the HOVON132 clinical trial protocol for inclusion of patients. Patient characteristics are described in supplementary table 1 and 2. Mutations and cytogenetics at time of diagnosis are described in supplementary figure 1.The median age of the cohort was 56.3 years, 13 patients were younger than 60 years, and 19 were older. There were 19 males and 13 females. The risk classification based on genetics were equally distributed with 11 favorable risk, 9 intermediate and 12 adverse risk patients. 12 of the patients were alive at 5 years.
Recruitment	Patients were consecutively included in this observational study, and samples were collected between 2014-2016 at Haukeland University Hospital, Bergen, Norway, and Oslo University Hospital, Rikshospitalet, Norway. The inclusion criteria were: newly diagnosed fit AML patients eligible for induction therapy (3+7) with cytarabine and daunorubicine/idarubicine. Patients were requited by their treatment responsible physician at Haukeland University Hospital or Rikshospitalet, Oslo. There were no self-selection bias in this study as patients were consecutively sampled in the time periode 2014-2016. No compensation was given to the patients who contributed to this study. A small fee was given to the healthy donors who volunteered to donate bone marrow samples to the study.
Ethics oversight	The study was performed in accordance with the Declaration of Helsinki, and all samples, including samples from healthy donors, were collected following written informed consent. The biobank and the clinical protocols were approved by the ethical committee at the University of Bergen (Ethical approval REK Vest 2012/2245, 2012/2247 and the Regional Committee for Medical Research Ethics South-East Norway (REK 2015/2012), the Norwegian Medicines Agency and The Data Inspectorate

Note that full information on the approval of the study protocol must also be provided in the manuscript.

Field-specific reporting

Please select the one below that is the best fit for your research. If you are not sure, read the appropriate sections before making your selection.

[x] Life sciences [ ] Behavioural & social sciences [ ] Ecological, evolutionary & environmental sciences

For a reference copy of the document with all sections, see nature.com/documents/nr-reporting-summary-flat.pdf

Life sciences study design

All studies must disclose on these points even when the disclosure is negative.

Sample size	Patients were consecutively included in this observational study, and samples were collected between 2014-2016 at Haukeland University Hospital, Bergen, Norway, and Oslo University Hospital, Rikshospitalet, Norway. The cohort consist of 32 AML patients (30 de novo and two secondary AML) that received standard “7+3” induction therapy. Additionally, we analyzed samples from 2 AML patients who received dose-reduced "7+3" treatment.
Data exclusions	As stated in the methods section the antibodies CD25 (IL-2R) and CD11b (Mac-1) were excluded as clustering channels due to large variability in staining between barcodes, not correctable by standardization. Intracellular marker p-4E-BP1 (T37/T46) was not included in the staining of barcode 5 and 6.
Replication	We have not replicated the results, however, we have validated our mass cytometry findings through different methodologies; computational by bi-axial gating strategy, Super-SILAC proteomics and RNAseq. All antibodies applied in this study was titrated on lyse/fix peripheral blood from 5 healthy donors, 9 of the patients included in this study and bone marrow from 5 healthy donors.
Randomization	Sample randomization was done when setting up the barcode pools for mass cytometry. Different diagnosis were distributed on the seven barcode batches. Each barcode included samples from both sample sites (Oslo and Bergen). The survival of the patients was not known when the samples were analyzed by mass cytometry, therefore responders and non-responders were distributed randomly between the different barcode batches.
Blinding	The FlowSOM clustering was done prior to the LASSO regression analysis, the choice of metacluster number was therefor done blinded. The different clusters were used as features in the LASSO regression analysis.

Reporting for specific materials, systems and methods

We require information from authors about some types of materials, experimental systems and methods used in many studies. Here, indicate whether each material, system or method listed is relevant to your study. If you are not sure if a list item applies to your research, read the appropriate section before selecting a response.

Materials & experimental systems

n/a	Involved in the study	
☐	☒	Antibodies
☒	☐	Eukaryotic cell lines
☒	☐	Palaeontology and archaeology
☒	☐	Animals and other organisms
☐	☒	Clinical data
☒	☐	Dual use research of concern

Methods

n/a	Involved in the study	
☒	☐	ChIP-seq
☒	☐	Flow cytometry
☒	☐	MRI-based neuroimaging

Antibodies

Antibodies used	Antibody panel: antibody, [phopho site] (Clone, catalog number)(Dilution): cleaved capsase-3 (D3E9, 3142004A)(1:400), p4EBP1 [T37/T46] (236B4, 3149005C)(1:400), pSTAT5 [Y694] (47, 3150005A)(1:400), pAkt [S473] (47, 3152005C)(1:200), pSTAT1 [Y701] (58D6, 3153003C)(1:400), pRB [S807/S811](J112-906, 413568)(1:400), p-p38[T180/Y182] (D3F9, 3156002C)(1:1600), pSTAT3 [Y705] (4/P-STAT3,3158005C)(1:400), pAxl [Y779] (Y779, MAB6965)(1:200), CyclinB1 (GNS-1, 3153009C)(1:200), pCREB [S133] (87G3, 3165009C)(1:200), pNFkB p65 [S529] (K10-895.12.50, 3166006A)(1:400), pERK 1/2 [T202/Y204] (D13.14.4E, 3167005C)(1:400), pS6 [S235/S236] (N7-548, 3172008C)(1:400), pHistone3 [S28] (HTA28,3175012A)(1:200), CD11b (Mac-1) (ICRF44,3209003B)(1:1600), CD8a (RPA-T8, 3162015C)(1:3200), CD33 (WM53, 3163023B)(1:800), CD34 (581, 343531)(1:12000), CD3 (UCHT1, 3170001B)(1:3200), CD123 (IL-3R) (6H6, 3151001B)(1:800), CD56 (NCAM) (B159, 3155008B)(1:400), CD14 (M5E2, 301843)(1:800), CD117 (c-kit) (104D2, 3143001C)(1:1600), CD38 (HIT2, 3144014C)(1:800), CD4 (RPA-T4, 3145001B)(1:800), CD64 (10.1, 3146006C)(1:400), CD16 (3G8, 3148004B)(1:200), CD66b (G10F5, 305102)(1:3200), CD20 (2H7, 3147001B)(1:400), CD90 (Thy-1) (5E10, 3159007C)(1:400), CD25 (IL-2R) (2A3, 3169003C)(1:3200), CD45 (HI30, 3089003B)(1:4000), Axl (1H12, Provided by BerGenBio ASA)(1:200), HLA-DR (L243, 3174001C)(1:800), CD7 (CD7-6B7, 343107)(1:3200). All Antibodies except AXL (1H12), pAxl, pRb, CD34, CD7, CD14 and CD66b were purchased through Fluidigm (Standard bio tools). pRb were purchased from BD BioScience, pAxl from R&D, CD34, CD66b, CD14 and CD7 were purchased from BioLegend and AXL (1H12) was provided by BerGenBio ASA.
Validation	All antibodies applied in this study was titrated on lyse/fix peripheral blood from 5 healthy donors, 9 of the patients included in this study and bone marrow from 5 healthy donors. In addition all antibodies have been validated by supplier (please refer to the manufacturers notes on their website). Several of the antibodies included in this study have been validated in Gullaksen et al. Cytometry part A, 2019. The pERK-1/2 (T202/Y204) antibody was validated by western blot on several of the patients in this study, with Molm13 treated with GM-CSF and UO126 as positive and negative control.

Clinical data

Policy information about clinical studies

All manuscripts should comply with the ICMJE guidelines for publication of clinical research and a completed CONSORT checklist must be included with all submissions.

Clinical trial registration	N/A
Study protocol	N/A
Data collection	Ethical approval REK Vest 2012/2245, 2012/2247 and the Regional Committee for Medical Research Ethics South-East Norway (REK 2015/2012)
Outcomes	Ethical approval REK Vest 2012/2245, 2012/2247 and the Regional Committee for Medical Research Ethics South-East Norway (REK 2015/2012)
